# Exploring Routes to Enhance the Calculation of Free Energy Differences via Non-Equilibrium Work SQM/MM Switching Simulations Using Hybrid Charge Intermediates between MM and SQM Levels of Theory or Non-Linear Switching Schemes

**DOI:** 10.3390/molecules28104006

**Published:** 2023-05-10

**Authors:** Andreas Schöller, H. Lee Woodcock, Stefan Boresch

**Affiliations:** 1 Faculty of Chemistry, Department of Computational Biological Chemistry, University of Vienna, Währingerstr. 17, A-1090 Vienna, Austria; 2 Vienna Doctoral School in Chemistry (DoSChem), University of Vienna, Währingerstr. 42, A-1090 Vienna, Austria; 3 Department of Chemistry, University of South Florida, 4202 E. Fowler Ave., CHE205, Tampa, FL 33620-5250, USA; hlw@usf.edu

**Keywords:** free energy, indirect thermodynamic cycle, non-equilibrium simulation

## Abstract

Non-equilibrium work switching simulations and Jarzynski’s equation are a reliable method for computing free energy differences, $\Delta A^{\mathrm{low}\to \mathrm{high}}$, between two levels of theory, such as a pure molecular mechanical (MM) and a quantum mechanical/molecular mechanical (QM/MM) description of a system of interest. Despite the inherent parallelism, the computational cost of this approach can quickly become very high. This is particularly true for systems where the core region, the part of the system to be described at different levels of theory, is embedded in an environment such as explicit solvent water. We find that even for relatively simple solute–water systems, switching lengths of at least 5 ps are necessary to compute $\Delta A^{\mathrm{low}\to \mathrm{high}}$ reliably. In this study, we investigate two approaches towards an affordable protocol, with an emphasis on keeping the switching length well below 5 ps. Inserting a hybrid charge intermediate state with modified partial charges, which resembles the charge distribution of the desired high level, makes it possible to obtain reliable calculations with 2 ps switches. Attempts using step-wise linear switching paths, on the other hand, did not lead to improvement, i.e., a faster convergence for all systems. To understand these findings, we analyzed the solutes’ properties as a function of the partial charges used and the number of water molecules in direct contact with the solute, and studied the time needed for water molecules to reorient themselves upon a change in the solute’s charge distribution.

## 1. Introduction

**Figure 1:**
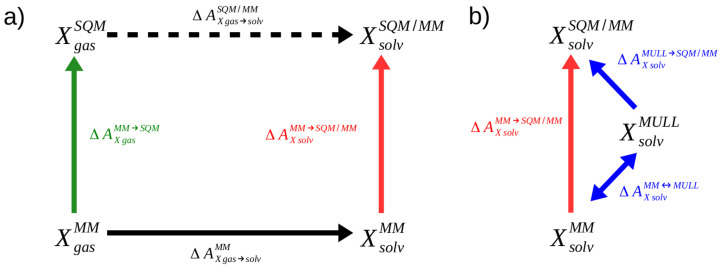
(**a**) Indirect alchemical thermodynamic cycle to compute $\Delta A_{X\;gas\to solv}^{SQM/MM}$ (dashed arrow) in three steps according to Equation (1). (**b**) Indirect alchemical thermodynamic cycle to compute $\Delta A_{X\;solv}^{MM\to SQM/MM}$ (red arrow) via hybrid charge intermediates; cf. Equation (8).

Since the first applications reported shortly before 1990 [1,2], so-called alchemical free energy simulations (FESs) have become an essential tool of computational chemists in academia and industry alike [3,4,5,6]. The free energy difference determines the spontaneities of chemical or biochemical processes, and hence, makes it possible to predict, e.g., the binding affinities of potential drugs [7,8,9]. In addition to computationally estimating an important macroscopic, thermodynamic quantity, FESs can also provide insight in the microscopic origins [10]. One source of error is the accuracy with which interactions within and between molecules are modeled in the underlying molecular dynamics simulations. In certain applications, force field-based descriptions may be insufficient [11]. Even if one is not interested in studying chemical reactions, the neglect of induced electronic polarization in classical force fields may lead to problems, even for seemingly simple applications, such as the computation of relative free energies of hydration of mono- or bivalent ions [12,13,14]. In such situations, hybrid quantum-mechanical/molecular mechanical (QM/MM) Hamiltonians are called for. However, even when using only semi-empirical quantum chemical methods (henceforth abbreviated as SQMs) for the core region, i.e., the part of the system that one is particularly interested in, the computational cost can quickly become prohibitive. Further, several tricks that are frequently used in alchemical FES do not work with SQM/MM Hamiltonians [15]. Early on, Warshel, Gao, and others pioneered the use of indirect cycles, also referred to as “multilevel” free energy simulations, as depicted in Figure 1a, which have become a widely used way of performing FES with an (S)QM/MM description of interactions [16,17,18].

To illustrate the indirect approach, we consider the calculation of the aqueous solvation free energy of a solute X. As can be seen in Figure 1a, the quantity of interest and the solvation free energy difference $\Delta A_{X\;gas\to solv}^{SQM/MM}$ at the high SQM/MM level of theory (dashed, black arrow in Figure 1a), can also be obtained in three steps according to

(1) $$\Delta A_{X\;gas\to solv}^{SQM/MM}=-\Delta A_{X\;gas}^{MM\to SQM}+\Delta A_{X\;gas\to solv}^{MM}+\Delta A_{X\;solv}^{MM\to SQM/MM}$$

In addition to computing the solvation free energy $\Delta A_{X\;gas\to solv}^{MM}$ at the force field (MM) level of theory (solid black arrow), two correction steps, accounting for the free energy difference of X in the gas phase and the aqueous solution between the two levels of theory (green and red arrows), are required. The development in this field over recent years shows that there is not only enormous potential, but also a high degree of interest in the FES community to use this strategy to compute free energy differences at the SQM/MM levels of theory [19,20,21,22,23,24,25,26,27].

Work by Heimdal and Ryde [28] made clear that the challenging step of indirect cycle SQM/MM FES is the calculation of the low-to-high corrections $\Delta A_{X\;gas}^{MM\to SQM}$ and $\Delta A_{X\;solv}^{MM\to SQM/MM}$: the green and red arrows of Figure 1a. Various approaches have been used, often requiring a sophisticated decomposition of the cycle and the employment of fitting procedures in order to be successful [18,29]. In the past, we showed that the corrections $\Delta A^{MM\to SQM}$ can be computed reliably and accurately using non-equilibrium work (NEW) methods [30,31,32,33,34,35]. In our work to date, computationally expensive protocols were used, which are unsuitable for most real-world applications. Recently, we optimized the NEW protocols regarding switching length and the number of switches, and presented a sufficiently efficient workflow for practical applications [36]. However, these optimized protocols were tested so far only in the gas phase, i.e., the green arrow of Figure 1a ($\Delta A_{X\;gas}^{MM\to SQM}$).

Here, we investigate whether the protocols suggested in Ref. [36] are also suitable for aqueous solution, i.e., when the region of interest is to be treated at a high level of theory, e.g., SQM, interacts with an MM environment ($\Delta A_{X\;solv}^{MM\to SQM/MM}$, red arrow in Figure 1a). In earlier work, we had noted that NEW switching simulations from a pure MM to an SQM/MM level of theory converged more slowly compared to gas phase transformations involving only MM to SQM, and observed that the charge distribution resulting from the fixed charges of the force field and the average charge distribution obtained at the SQM/MM level of theory were noticeably different [31]. We speculated that the necessary reorientation of water in response to such a change in the charge distribution of the solute might be responsible for the slower convergence. Using equilibrium methods to compute $\Delta A_{X\;solv}^{MM\to SQM/MM}$, Ito and Cui reported that inserting an intermediate state with fixed charges more similar to the charge distribution at the SQM/MM target state improved convergence considerably [37].

The solvent-reorientation dynamics of water in response to charge changes is experimentally well characterized and understood [38,39]. Two relaxation times, one faster than 0.5 ps and the other approximately 2 ps, can be discerned. Therefore, if the charge distribution of the region of interest is significantly different at the two levels of theory, NEW switching simulations of only 2 ps, as used in Ref. [31], may be too short to obtain converged results. With this in mind, we tested two strategies. (i) Similarly to the work by Ito and Cui, we introduce an intermediate state, which is still purely MM, but with the atomic partial charges modified to resemble the average charge distribution of the SQM description at the high level of theory. (ii) Since water reorientation in response to a change in charge distribution occurs on two timescales, the linear switching protocols we have used so far may be suboptimal. In particular, we test whether protocols in which switching is carried out initially at a faster rate, e.g., switching from the MM to a 35% SQM/MM description at just 10% of the switching length, followed by a second slower phase during the remaining 90% of the switching length, might lead to improved convergence. Obviously, both strategies can be combined. Model calculations exploring these two approaches are complemented by a series of analyses in which we investigate the effects of a solute’s charge distribution on properties such as its dipole moment, as well as on the interactions with surrounding waters.

The focus of this work is on the effect by which the charge distribution of a solute described at different levels of theory has on the convergence of calculating $\Delta A_{X\;solv}^{MM\to SQM/MM}$. To avoid unrelated complications from too-flexible molecules, where conformational preferences might be different at the low and high levels of theory, respectively, we mostly chose rather rigid model compounds for the test calculations. Rather than using (a subset of) the so-called “HiPen” test set [34], we selected tautomeric pairs (cf. Methods). Most of the compounds are rather rigid without rotatable bonds. Therefore, the pure MM and the SQM/MM descriptions of the systems—the solute (tautomer) are described by SQM, and all waters remain classical—differing primarily by the charge distributions of the solute at the two levels of theory.

The remainder of the manuscript is organized as follows. In Results, we first study the convergence of NEW switching simulations, using the optimized protocol from our preceding study [36] (switching lengths of 2 ps, 200 NEW switches per transformation). We then investigate how convergence $\Delta A_{X\;solv}^{MM\to SQM/MM}$ improves when employing switching lengths of 5 ps, which, however, may be too costly for many applications. Next, we present results using the two mitigation strategies outlined above. Our findings are rationalized using additional data characterizing the differences in solute properties, in particular the dipole moment at the two levels of theory, as well as details on the solvent-reorientation dynamics of water under the simulation conditions. In Section 4, we summarize the theoretical background, introduce the model systems, and provide the technical details of all simulations and analyses carried out.

## 2. Results

### 2.1. Overview of Calculated Free Energy Differences and Paths

Below, several approaches to calculate the free energy difference between two levels of theory are compared. To specify the method and the exact meaning of a free energy difference, we adopt the following labeling scheme:

$$\Delta A_{Xphase}^{level\;of\;theory\;I\;\to /↔\;level\;of\;theory\;II}$$

Here, the subscript *X* indicates the compound; the qualifier *phase* can either be “gas” (gas phase) or “solv” (solvated phase). In the superscript, *level of theory I* and *level of theory II* can be “MM”, “MULL(gas)”, “MULL(solv)”, “MULL(solv*)”, or “SQM(/MM)”. MM indicates the use of the regular force field. The abbreviation SQM indicates that the SCC-DFTB semi-empirical method was used to compute interactions, either for the full system (the gas phase), or for the solute (the aqueous solution; waters were always described classically). The labels MULL(gas), MULL(solv), and MULL(solv*) refer to three methods used to derive the average Mulliken charges described in Section 4.3.1.

A simple arrow → stands for a one-sided method to compute the free energy difference; in this work, this always means Jarzynki’s equation (JAR) [40]. The double-pointed arrow ↔ indicates the use of a two-sided method. As described in Section 4, we used Crooks’ equation (CRO) [41] to compute the reference results. Furthermore, the free energy differences between MM and the intermediate state with modified partial charges, i.e., MULL(gas), MULL(solv), or MULL(solv*), were computed using Bennett’s acceptance ratio method (BAR) [42].

As in our previous work, all results are reported relative to a reference result; i.e., instead of reporting, e.g., $\Delta A_{Xphase}^{MM\to SQM}$, we report the corresponding double free energy difference $\delta \Delta A_{Xphase}=\Delta A_{Xphase}^{MM\to SQM}-\Delta A_{Xphase}^{\mathrm{CRO}{}_{\mathrm{Ref}}}$. In the past, the reference results $\Delta A_{Xphase}^{\mathrm{CRO}{}_{\mathrm{Ref}}}$ were obtained with CRO, based on 200 instances of 2 ps forward and backward NEW switches. As described in Section 2.2 below, we decided to use the CRO results obtained from 5 ps switches as the reference (CRO${}_{\mathrm{Ref}}$). The exact definition of $\delta \Delta A_{Xphase}$ depends on whether the free energy difference between two levels of theory is computed in a single step, i.e.,

(2a) $$\delta \Delta A_{Xphase}=\Delta A_{Xphase}^{\mathrm{MM}\;\to /↔\;\mathrm{SQM}}-\Delta A_{Xphase}^{\mathrm{CRO}{}_{\mathrm{Ref}}}$$

or via an hybrid charge intermediate state. Here, we have

(2b) $$\delta \Delta A_{Xphase}=\Delta A_{Xphase}^{\mathrm{MM}\;↔\;\mathrm{MULL}}+\Delta A_{Xphase}^{\mathrm{MULL}\;\to \;\mathrm{SQM}}-\Delta A_{Xphase}^{\mathrm{CRO}{}_{\mathrm{Ref}}}$$

to account for the correction step between the MM representation and the hybrid Mulliken charge intermediate state; cf. Equation (8).

### 2.2. Performances of 2 and 5 ps Linear Switching Protocols in Solution

Before carrying out calculations in aqueous solution, the optimized JAR protocol from Ref. [36] was tested for all compounds in the gas phase. All results can be found in Table S3 of SI. The model systems used in this work are described in Section 4.2. Except for **1-t1** (see further down), $\left|\delta \Delta A\right|$ was <0.1 kcal/mol. While δΔA(1-t1)≈0.35 kcal/mol is slightly larger than the ideal maximum deviation of ±0.25 kcal/mol, this value is still acceptable for most practical applications. Thus, the protocol recommendations of our previous study [36] are applicable to the model compounds studied here. Based on the gas phase results, any noticeable deterioration of δΔA therefore has to be caused by differences in the description of solute–solvent interactions at the two levels of theory.

**Figure 2:**
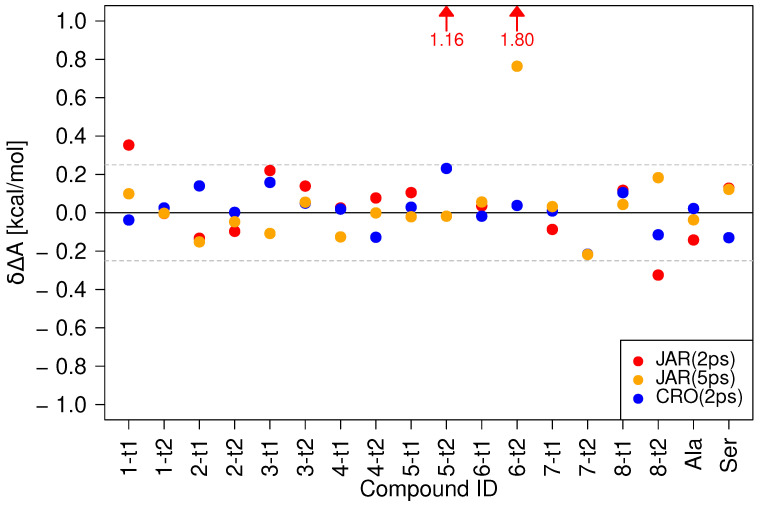
Comparison of $\delta \Delta A_{Xsolv}^{MM\to SQM}$ using three protocols without intermediate state: JAR(2ps): 2 ps forward switches from MM to SQM, JAR(5ps): 5 ps forward switches from MM to SQM, and CRO(2ps): 2 ps forward and backward switches. In all cases, the CRO results obtained from 5 ps switches were considered as the reference values CRO${}_{\mathrm{Ref}}$. For **5t-2** and **6t-2**, the JAR(2ps) results are off-scale, which is indicated by the red arrows.

In Figure 2, the δΔA values for all compounds obtained via the three protocols/methods are compared against the reference results obtained from 200 forward/backward switches of 5 ps length: 200 MM to SQM switches of 2 ps length, the recommended protocol from Ref. [36], (JAR(2ps), red circles), 200 MM to SQM switches of 5 ps length (JAR(5ps), orange circles), and the CRO results obtained from 200 forward/backward switches of 2 ps length (CRO(2ps), blue circles). The data visualized in Figure 2 are tabulated in Table S2; even more detailed results can be found in Tables S4 and S5. For the shortest protocol, JAR(2ps), four results lie outside the ±0.25 kcal/mol threshold indicated by the two dashed lines in the figure. Taking error bars into account (see Table S2), the results for **1-t1** and **8-t2** can be considered as acceptable, $\left|\delta \Delta A\right|<0.4$ kcal/mole; furthermore, δΔA (**1-t1**) is comparable to the value obtained in the gas phase (see Table S3). However, for **5-t2** and **6-t2**, the deviation from the reference result is larger than 1 kcal/mol and would lead to a sizable systematic error.

The use of a 5 ps switching length (JAR(5ps), orange circles) certainly helps, but one poor result, **6-t2**, δΔA≈0.8 kcal/mol, remains. Thus, the relatively inexpensive protocol suggested in Ref. [36] cannot be recommended for calculations in solution. Even worse, the use of more costly switching simulations of 5 ps length does not help in all cases.

Figure 2 also shows results for the CRO(2ps) protocol (blue circles). Compared to our findings in the gas phase [36], the differences to the reference result CRO(5ps) are noticeably more pronounced. Furthermore, as can be seen in Tables S4 and S5, the statistical uncertainty of the CRO(2ps) results was consistently higher than those of the CRO(5ps) results, even though the overlap between the forward and backward work distributions seemed adequate in all cases. While all CRO(2ps) δΔA values lie within the ±0.25 kcal/mol threshold, we nevertheless decided to choose CRO(5ps) as the reference protocol.

Three of the four compounds failing the ±0.25 kcal/mol threshold using the JAR(2ps) protocol, **5-t2**, **6-t2**, and **8-t2**, are lactams. Interestingly, the corresponding tautomeric lactim states do not cause any problems. The pair **1-t1**/**1-t2** belongs to the keto-enol class of tautomerism. For **1-t1**, δΔA≈0.35 kcal/mol in both the gas phase and in aqueous solution; therefore, different conformational preferences at the two levels of theory may be responsible for the poor convergence when using switching lengths of 2 ps.

### 2.3. Performances of Hybrid Charge Intermediates

**Figure 3:**
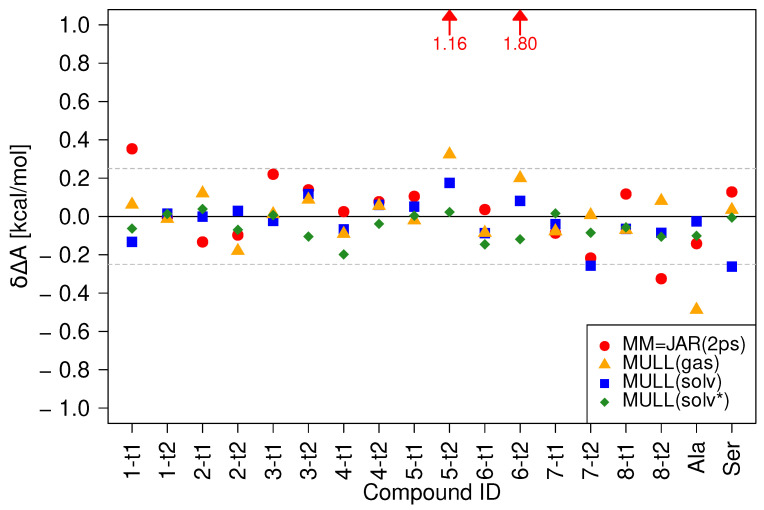
Comparison of δΔA obtained with three hybrid intermediate charge states: MULL(gas), orange triangles; MULL(solv), blue squares; MULL(solv*), green diamonds. All results were obtained from 200 NEW switches of 2 ps length to compute $\Delta A^{\mathrm{MULL}\to \mathrm{SQM}}$ and include the correction $\Delta A^{\mathrm{MM}↔\mathrm{MULL}}$; cf. Equation (2b). The JAR(2ps) results already shown in Figure 2 (red circles) are included for comparison purposes.

In Figure 3, we summarize the performances of the indirect NEW switching protocols using a hybrid intermediate charge state in terms of δΔA, cf. Equation (2b). As described in Methods (Section 4.3.1), three approaches to obtain average Mulliken charges were tested: averaging over gas phase configurations (MULL(gas), over configurations sampled in aqueous solution at the MM level of theory (MULL(solv)), and over configurations sampled in aqueous solution at the SQM/MM level of theory (MULL(solv*)). The results obtained with the direct JAR(2ps) protocol (red circles), already shown in Figure 2 and henceforth referred to as just MM, are included as well.

Clearly, all three protocols employing an intermediate hybrid charge state perform much better than the direct NEW switches (MM), though not to the same degree. Using the MULL(gas) intermediate state (orange triangles), the ±0.25 kcal/mol threshold for **5-t2** is still missed, δΔA=0.32 kcal/mol, and the result for **Ala** becomes worse (δΔA=−0.49 kcal/mol). For MULL(solv), the blue squares, all results lie de facto within the ±0.25 kcal/mol threshold (δΔA(5-t2)=−0.28 kcal/mol, δΔA(Ser)=−0.26 kcal/mol). It should be noted that taking the statistical uncertainty of the results into account, none of these deviations from the ±0.25 kcal/mol threshold are statistically significant. Finally, all MULL(solv*) results (green diamonds) lie well within the ±0.25 kcal/mol threshold.

Figure 3 shows that in most cases, the use of a hybrid intermediate charge state lowers δΔA, and typically, the use of MULL(solv*) leads to the best results, followed in this order, using MULL(solv) and MULL(gas). To quantify this, we report in Table 1 the mean absolute deviation (MAD) for the data shown in Figure 3, together with the spread of δΔA for each of the protocols. Already, the use of MULL(gas) lowers the MAD from 0.29 to 0.12 kcal/mol, and the largest error δΔA reduces from 1.80 to 0.49 kcal/mol. The MULL(solv) intermediate state leads to a further improvement (MAD=0.09, and the largest error is 0.28 kcal/mol). Finally, the MAD for MULL(solv*) is 0.07 kcal/mol and the largest δΔA is as low as 0.20 kcal/mol.

The improvements resulting from the hybrid intermediate charge states are roughly inversely proportional to the computational effort to obtain the average Mulliken-like charges. The MULL(gas) and MULL(solv) charges are obtained from configurations at the MM level of theory; the required computations at the SQM(/MM) level of theory to obtain the Mulliken charges are slightly more costly for the solvated system. By contrast, to obtain the MULL(solv*) charges, sampling needs to be carried out at the high (SQM/MM) level of theory. Given that the improvements of using MULL(solv*) rather than MULL(solv) are relatively small, this leads to an additional computational cost that, at least for the systems studied here, seems not to be worthwhile. In other words, the use of the MULL(solv) hybrid partial charges seems to be a good compromise between correctness and computational cost. Each of the indirect NEW switching protocols requires the calculation of $\Delta A^{\mathrm{MM}↔\mathrm{MULL}}$; however, this is a strict force field-based free energy calculation, which can be computed quickly and efficiently on GPUs using the CHARMM/OpenMM interface (cf. Section 4.4.1).

### 2.4. Performances of Modified Switching Protocols

**Figure 4:**
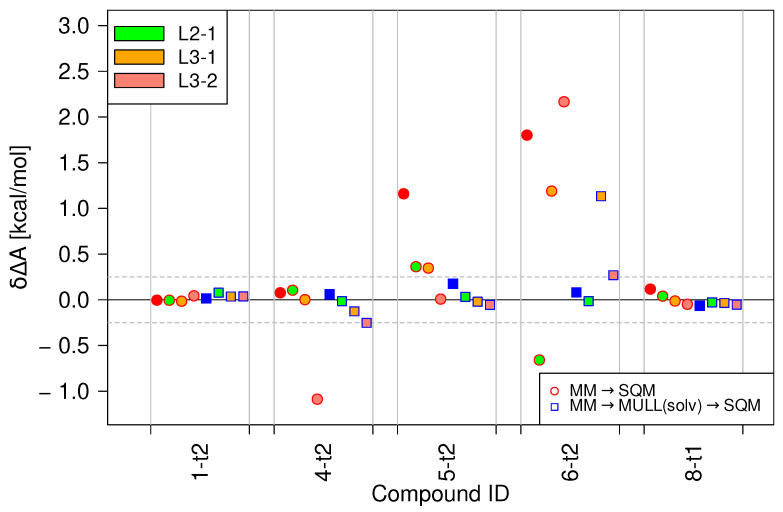
Performances of the three modified switching protocols for a subset of the compounds. Results for the direct NEW switching protocols MM → SQM are shown as red circles; the results for calculations using the MULL(solv) hybrid charge intermediate state are blue squares. The fill color of the circles/squares indicates the stepwise linear switching protocol used (green = **L2-1**, orange = **L3-1** and salmon = **L3-2**). For comparison purposes, the MM/JAR(2ps) (red circles) and MULL(solv) results (blue squares), both using the default switching protocol **L1**, are included as well.

Figure 4 summarizes the results obtained with stepwise linear switching protocols for a subset of the model compounds. For details, see Section 4.3.2 and Figure 11. In all cases, the baseline results, indicated as red, solid circles, are the free energy difference obtained from one-step MM → SQM switches, i.e., the MM/JAR(2ps) results already presented. For the first problematic case, **5-t2**, all three stepwise linear protocols reduce δΔA considerably. However, in the case of **6-t2**, it is difficult to speak of improvements. Two protocols, **L2-1** and **L3-1**, lower δΔA, but the values are still far outside of the ±0.25 kcal/mol threshold. The third protocol, **L3-2**, which worked extremely well for **5-t2**, actually increases δΔA for **6-t2** compared to **L1**, the default linear protocol. The other three compounds included in the subset were chosen as negative controls because they already performed well with the JAR(2ps) protocol. The performance of the stepwise linear protocol is comparable, except for one poor result for **4-t2** when using **L3-1**.

Turning to the combination of stepwise linear and indirect NEW switching protocols with the MULL(solv) hybrid intermediate charge state (shown as squares), most of the results lie within the ±0.25 kcal/mol threshold. However, since in this case, the performance of the linear protocol was excellent to begin with, any improvements are marginal. Moreover, for **4-t2**, the stepwise linear protocols perform slightly poorer, and for **6-t2**, δΔA obtained with the **L3-1** is >1 kcal/mol.

Based on these findings, the performances of the stepwise linear switching protocols seems mixed at best. None of the protocols tested consistently improved the results. Furthermore, even when the results were improved and fell within the ±0.25 kcal/mol threshold, the standard deviation of the work values, $\sigma_{W}$, was always larger than for the linear switching protocol. Since $\sigma_{W}$ is a sensitive indicator of whether one can expect the convergence of JAR calculations [32], the utility of the protocols that increase it seems doubtful.

### 2.5. A Detailed Analysis of the Factors Affecting Convergence

#### 2.5.1. Effects of Charge Distribution on Solute Properties

To quantify the differences between the various charge models used in this study, we calculated the root-mean-square deviation of the atomic charge differences ${\mathrm{RMSD}}_{q}$ between the MM, MULL(gas), MULL(solv) representations, using the MULL(solv*) charges as the reference value (see Section 4.3.3, Equation (9)). Since the latter are the average of the Mulliken charges derived from simulations at the SQM/MM level of theory, they are closest to the fluctuating charge distribution at the target high level of theory. The variability of the Mulliken charges derived from the SQM(/MM) reevaluations about their mean values were always extremely low, fluctuating typically less than ±0.03 e about the mean; the largest value observed was 0.08 e. These low standard deviations make the use of average values meaningful in the first place. In addition to ${\mathrm{RMSD}}_{q}$, we looked at the magnitude of the differential dipole moment $\left|\Delta \vec{\mu }\right|=\Delta \mu$ (Equation (10)) and the angle $\Theta_{\mathrm{SQM}}$ between the dipole moment of a charge distribution and that of the MULL(solv*) reference charges (Equation (11)).

The individual results for each compound are listed in Table S12 and depicted in Figures S4–S6 of SI. Some overall trends can be seen from the MAD values of ${\mathrm{RMSD}}_{q}$, Δμ, and $\Theta_{\mathrm{SQM}}$ in Table 2. As expected, the MAD(${\mathrm{RMSD}}_{q}$) is largest for MM and smallest for MULL(solv), with MULL(gas) in between; the same is the case for MAD(Δμ). This is in accord with the MAD(δΔA) results of Table 1 in Section 2.3, where the use of the MULL(gas) intermediate charge state led to a noticeable improvement, and the MULL(solv) charges lowered the MAD(δΔA) even further. The MAD($\Theta_{\mathrm{SQM}}$) behaves somewhat differently; here, the MULL(gas) value is lower than that of MULL(solv). As can be seen in Table S12 and Figure S6, this holds true for most compounds. Nevertheless, $\Theta_{\mathrm{SQM}}$ of MULL(solv) is always smaller than that of the MM charges. The findings strongly suggest that the differences between the charge distribution at the two levels of theory have a strong influence on the convergence of the calculations of $\Delta A^{\mathrm{high}\to \mathrm{low}}$ via JAR, with the difference in magnitude of the two dipole moments being more important than the orientation of the respective dipole moment vectors.

Based on the overall convergence results (Figure 2), we surmised that compounds containing a lactam moiety tended to converge more slowly than the corresponding lactim state, e.g., **5-t2** vs. **5-t1**, etc. One reason for this is that the differences between the MM and the SQM(/MM) descriptions are greater for the lactams than for the lactims. The analysis of partial charges points in this direction as well. Several differences can be discerned in the detailed charge distribution data for the individual molecules, e.g., Figure S4. For all lactim–lactam pairs (compounds **2**, **3**, **4**, **5**, **6**, **8**),the MM ${\mathrm{RMSD}}_{q}$ of the lactam state is noticeably higher than that of the corresponding lactim state. Similarly, the MM ${\mathrm{RMSD}}_{q}$ of each of the lactams (red circle) is at least 0.1e higher than for MULL(solv) (blue squares).

**Figure 5:**
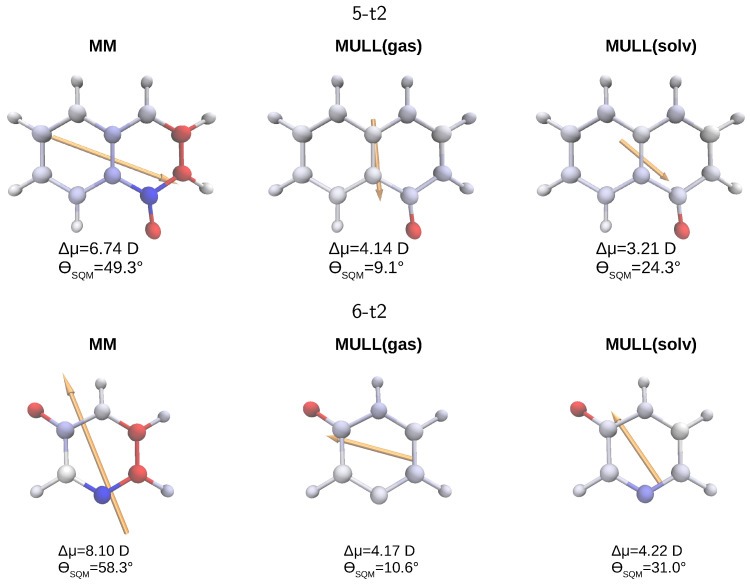
Graphical representation of differences in partial atomic charges compared to MULL(solv*) for **5-t2** (**top**) and **6-t2** (**bottom**). Charge differences are indicated as a color gradient from blue (δq=−0.2e) to red (δq=+0.4e). The differential dipole moment $\Delta \vec{\mu }$ is displayed as an orange arrow. The magnitude $\Delta \mu =\left|\Delta \vec{\mu }\right|$ and the angle $\Theta_{\mathrm{SQM}}$ between the dipole moment of the respective charge distribution with that of the MULL(solv*) reference distribution are given below each structure.

In Figure 5, we visualize some points made above for the two molecules, for which obtaining a converged $\Delta A^{\mathrm{MM}\to \mathrm{SQM}}$ was most difficult, **5-t2** (top) and **6-t2** (bottom). For each of the three charge representations, MM, MULL(gas), and MULL(solv), we display the differential atomic charges, both as labels, as well as a color gradient from blue to red, and the resulting differential dipole moment vectors (orange arrows). The magnitude of the differential dipole moment $\Delta \mu =\left|\Delta \vec{\mu }\right|$ and the angle $\Theta_{\mathrm{SQM}}$ between the dipole moment of a charge distribution and that of the MULL(solv*) reference charge distribution are listed directly. The difference in partial charges and the MULL(solv*) charges can be large, e.g., the difference for the -C=O part of the lactam moiety is almost ±0.5e for **5-t2** (top, left in Figure 5). One further sees that the charge differences become smaller between the MM and the two MULL charge sets, with atoms colored in clear blue or red for MM; e.g., the -C=O group (left side) has just a shade of blue or red for MULL(solv) (right side). Accordingly, the length of the orange arrows, i.e., Δμ of the MULL charge states, is noticeably smaller than that for MM. Even more detailed plots, including the exact values of the charge difference for each atom, for **3-t2**, **4-t2**, **5-t2**, **6-t2**, and **8-t2**, can be found in Figures S7–S11.

#### 2.5.2. Effects of Charge Distribution on the First Solvation Shell

**Figure 6:**
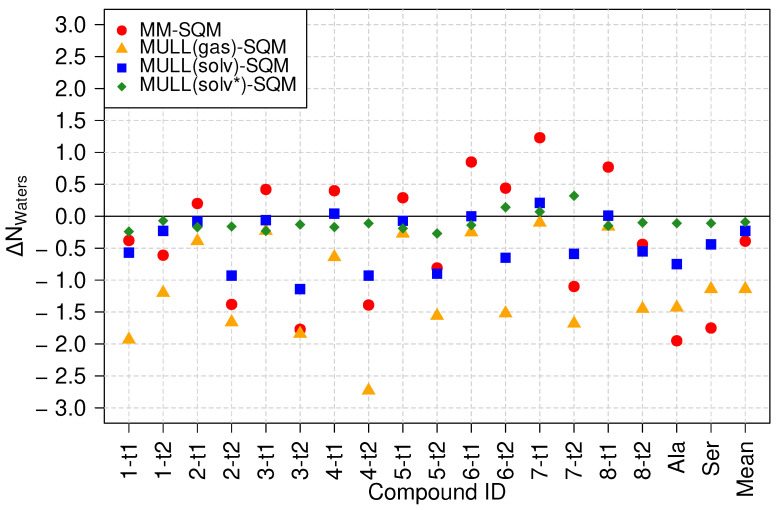
Difference in the average number of water molecules closer than 3 Å, compared to SQM/MM. The average number of water molecules in the SQM/MM simulations was used as the reference value.

Figure 6 displays the difference between the average number of waters $\Delta N_{Waters}$ within ≤3 Å of the solute. The numbers are relative to the target high-level state, i.e., the average number of waters found in the SQM/MM simulations. First, one sees that $\Delta N_{Waters}$ for the MULL(solv*) charges (green diamonds), which were also derived from the SQM/MM simulations, are very close to zero, in accord with expectation. Several results for the MULL(solv) charges (blue squares) are also quite close to zero, but mostly for the lactams (particularly **2-t2**, **3-t2**, **4-t2**, **5-t2**) the difference in water molecules $\Delta N_{Waters}\approx -1$. For the MULL(gas) charges (orange triangles), all $\Delta N_{Waters}$ values are negative, i.e., on average, there are fewer water molecules in close contact with the solute, compared to SQM/MM. This is not too surprising, because the charges were derived from SCC-DFTB gas phase calculations; hence, the solute did not experience polarization from its interaction with the solvent. This may also explain why on average, the use of the MULL(gas) hybrid intermediate state performed worse than MULL(solv), even though it performed significantly better than MM (cf. Table 1).

The MM results (red circles) fall into two distinct groups, with either more or less water present than in SQM/MM. For most lactams, with the slightly surprising exception of **6-t2**, $\Delta N_{Waters}$ is negative, whereas the lactims, i.e., have a positive $\Delta N_{Waters}$. Thus, one sees again distinct differences in properties for the force field representations of lactims and lactams, respectively.

#### 2.5.3. Water Reorientation Dynamics

**Figure 7:**
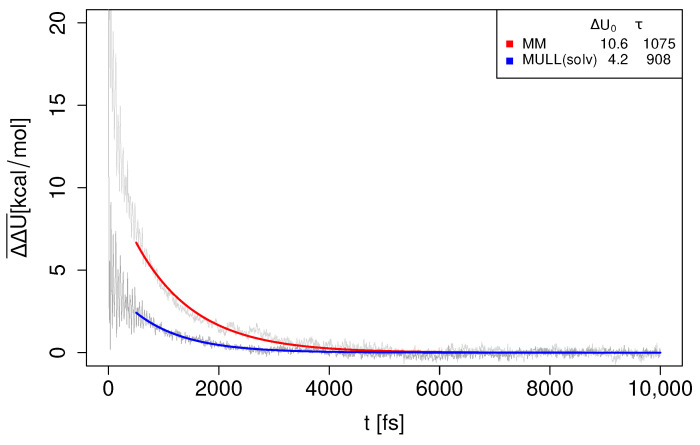
Water reorientation dynamics of TIP3P solvent for **5-t2** when switching from MM and MULL(solv) to SQM/MM. Raw data are shown in gray; fit for MM in red and fit for MULL(solv) in blue. The fit parameters ($\Delta U_{0}$ in kcal/mol, τ in fs) are listed in the inset; cf. Equation (14).

In the previous subsections, we characterized the influences of different charge distributions (atomic partial charges) on the static properties of the solute and the surrounding solvent layer in several ways. As the endpoints of the NEW switches have different charge distributions, it is of considerable interest to study the dynamics of solvent reorientation. We therefore computed the unnormalized Stoke shift relaxation $\bar{\Delta \Delta U(t)}$ for several compounds, and the MM and MULL(solv) charge distributions (see Section 4.3.3, Equation (13)). The results for **5t-2** are shown in Figure 7; analogous plots for **4t-2**, **6t-2**, and **8t-2** can be found in Figures S12–S14 in SI. All fit parameters are summarized in Table S13 of SI.

As described in Section 4.3.3, the mono-exponential fit was carried out in such a way that it picks out the slow process(es) of water reorientation. Looking at Figure 7, one sees that the relaxation times for MM and MULL(solv) are relatively comparable (τ≈1 ps), but that the prefactor $\Delta U_{0}$ is quite different (>10 kcal/mol for MM and ≈4 kcal/mol for MULL(solv)). Thus, while $\bar{\Delta \Delta U(t)}$ has for all practical reasons reached 0 after about 3 ps in the MULL(solv) case (blue curve), for MM (red curve), this is the case only after 5 ps. Since we would ideally carry out NEW switches of only 2 ps length, the obvious question is the value of ΔU(t=2ps). Using the fit parameters, we find that MM ≈ 1.7 kcal/mol, and that MULL(solv) ≈ 0.5 kcal/mol. These values roughly mirror the values for δΔA for MM/JAR(2ps) and MULL(solv), respectively.

For the second problem case, **6-t2**, very similar results were obtained; see Figure S13 and Table S13.

Overall, the slow decay process of $\bar{\Delta \Delta U(t)}$ always occurs with a time constant τ≈1 ps. Thus, the degree to which the system is still out of equilibrium regarding the SQM/MM target state therefore depends crucially on how big the difference is at t=0. Thus, it becomes clear that convergence is facilitated if the solute charge distributions of the initial and final state resemble each other, explaining why better results were obtained for all the MULL intermediate charge states. Phrased differently, to obtain converged JAR results from 2 ps switching simulations, the charge distributions of the initial and final states have to be very similar. Since the determining factor seems to be the initial difference in charge distribution, using stepwise linear switching paths cannot help much, explaining at least in part why we did not obtain any real improvements from the stepwise linear protocols we attempted.

#### 2.5.4. Interplay between Charge Distribution and Conformational Preferences

**Figure 8:**
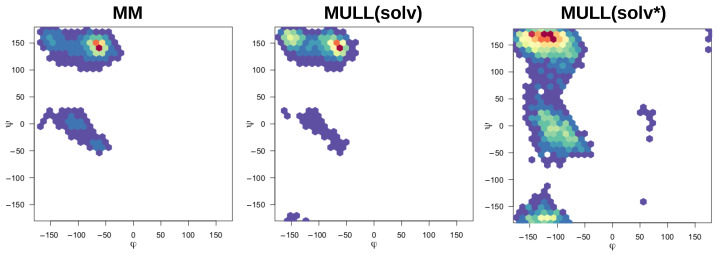
Ramachandran plots of pseudo-dipeptide/blocked alanine.

**Figure 9:**
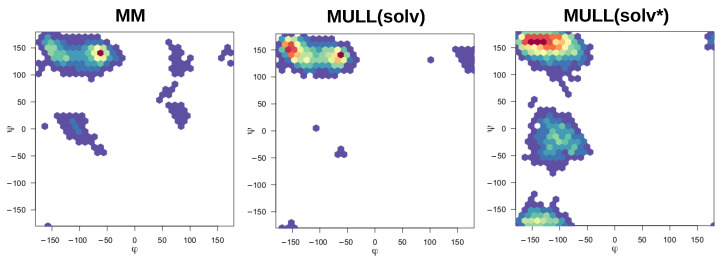
Ramachandran plots of pseudo-dipeptide/blocked serine.

The compounds used in this work were mostly rigid and specifically chosen to avoid complications from conformational degrees of freedom, the exceptions being **1-t1** and the blocked amino acids **Ala** and **Ser**. Modifying partial charges as for the MULL hybrid intermediate states may have an effect on conformational preferences. As can be seen in Figure 3, all three MULL hybrid intermediate states improve the convergence for **1-t1**. For the two blocked amino acids, MULL(gas) results in a poorer convergence for **Ala**, and MULL(solv) results in a slightly poorer convergence for **Ser**. Given that **Ala** and **Ser** are the smallest possible peptide-like molecules with protein backbone-like ϕ and ψ dihedral angles, we performed some analyses on the dihedral angle conformational preferences. In earlier work [31], we showed that purely classical Hamiltonians and SQM/MM Hamiltonians resulted in different preferred conformations of ϕ and ψ, as well as $\chi_{1}$ in the **Ser** case. In Figure 8 and Figure 9, ϕ/ψ maps for **Ala** and **Ser** are shown for MM, MULL(solv), and SQM. The differences between MM (left) and SQM (right) are clearly visible. For both blocked amino acids, MM has a single narrow minimum at $\phi \approx 150^{\circ }$, $\psi \approx -50^{\circ }$, whereas SQM has a broader distribution at $\phi \approx 150^{\circ }$, and $-150^{\circ }<\psi <-50^{\circ }$. While the MULL ϕ/ψ maps (middle plots) are more similar to MM than to SQM, a second minimum at $\phi \approx 150^{\circ }/\psi \approx -150^{\circ }$ has appeared. Thus, although the effect is small, the use of hybrid charge intermediates also makes this state slightly more similar to a high-level state, in terms of conformational preferences.

## 3. Discussion

The optimized protocol proposed in Ref. [36] (200 switches of 2 ps length) would be efficient enough for many practical applications. However, it has only been validated in the gas phase. If water reorientation is responsible for the slower convergence in aqueous solution, as observed previously [31,37], then, given the experimentally known slower relaxation time constant of >1 ps, switching lengths of 2 ps may not be long enough. This suspicion was confirmed in this work, and we found that in one case, even a switching length of 5 ps was insufficient.

We explored two approaches to keep the switching length at 2 ps: (i) introducing a hybrid intermediate charge state, which makes the solute charge distribution more similar to that at the SQM/MM level of theory. (ii) Since the solvent reorientation also has an ultrafast component, we used a stepwise linear switching scheme instead of the standard one, switching faster, initially followed by one or two slower steps. While the use of hybrid intermediate charge states significantly improved convergence, the benefits of multi-stage switching protocols were mixed.

The partial charges of the intermediate hybrid states were obtained by averaging over the Mulliken charges obtained from 200 energy evaluations at the SQM level of theory. The computational cost of this is relatively small; the overhead, if any, comes from sampling the configurations over which one averages in the first place. Not surprisingly, the best results (lowest overall δΔA; see Table 1) were obtained from coordinate sets generated during SQM/MM simulations, i.e., calculations at the target high level of theory (MULL(solv*)). While we have performed such calculations to obtain reference results with CRO, we would ideally prefer to avoid them in practical applications. In contrast, the cheapest approach is to use the coordinate sets saved during the MM gas phase simulations (MULL(gas)). While these results were an improvement over the direct MM→SQM/MM switches, the MAD(δΔA) was higher than that of MULL(solv*). The results for the third method tested, MULL(solv), fell in between. Here, the configurations to be reevaluated at the SQM/MM level of theory were generated from the MM simulations in the solvated phase. Since one can reuse the stored coordinates/restart files to start the NEW switching simulations, the computational cost is practically zero. All results obtained with the MULL(solv) hybrid intermediate charge state met our rather stringent quality criteria; the improvements over the direct MM→SQM/MM switches were noticeable, and the difference in convergence over the more expensive MULL(solv*) approach was small (a MAD of 0.09 instead of 0.07 kcal/mol). Thus, the MULL(solv) approach to derive an intermediate charge representation seems to be a good compromise between computational cost and convergence improvement. While for this study, averaged Mulliken charges were the logical choice because of the use of SCC-DFTB, other charge assignment procedures could be explored if different QM methods were used, such as ESP or CM5-symmetrized charges [19,43]. All methods require an additional, free energy simulation to obtain $\Delta A^{\mathrm{MM}↔\mathrm{MULL}}$ at the low level of theory, but its computational cost is small compared to that of the NEW switching simulations needing SQM/MM Hamiltonians.

As reported in Section 2.4, we have not seen consistent convergence improvements for all systems using the two-stage and three-stage switching protocols. This prompted a detailed analysis of how the MM and SQM/MM representations differ. Comparing the MM charges of the force field to the average Mulliken charges, one sees that there are indeed surprisingly large differences in charge distribution, and hence, the solute dipole moment. The differences in properties of the solute have a noticeable effect on the number of water molecules in close contact with the solute. Furthermore, by studying the time correlation function $\bar{\Delta \Delta U(t)}$ after switching from one level of theory to the other, we were able to confirm the time scales of experimental solvation spectroscopy. There clearly is a fast process with a relaxation time ≪1 ps, and a slower process with τ≈1 ps. This slower time constant does not change much if the initial charge distributions are equal to MM or MULL(solv). If the difference between the initial and final distribution is large, then the effect of the unfinished solvent reorientation after 2 ps (the switching length we are aiming for), will also be large. If the two charge distributions are more similar, as is the case for MULL(solv), then ΔU(t=2ps) is small enough to have little effect on the results, even though the solvent reorientation may not be complete. On the one hand, this observation explains the effectiveness of using hybrid charge intermediate states. On the other hand, it helps to rationalize our failure to obtain consistently convergent results with the multistage switching schemes. While one could certainly construct more complex switching paths, the speed of the solvent reorientation would remain the same.

## 4. Materials and Methods

### 4.1. Computing Free Energy Differences between the Levels of Theory Using NEW Methods

In several previous studies, [30,31,32,33,34,35,36] we demonstrated that NEW methods are a robust means of computing free energy differences between levels of theory, such as an MM and an SQM/MM description of a system. Given a series of non-equilibrium work $W^{MM\to SQM/MM}$ values obtained from switching simulations, the free energy difference is computed according to Jarzynski’s equation [40]:

(3) $$\Delta A^{MM\to SQM/MM}=-k_{B}T\ln\langle \exp(-\beta W^{MM\to SQM/MM})\rangle$$

where $k_{B}$, *T*, and β have the usual meanings of Boltzmann’s constant, absolute temperature, and $\beta =1/k_{B}T$. The angular brackets indicate averaging over a sufficiently large number of individual work values. To realize this scheme, one has to save snapshots during an equilibrium MD simulation at the MM level of theory. These serve as the starting point for the switching simulations using a hybrid energy function:

(4) $$U\left(\lambda_{t}\right)=\left(1-\lambda_{t}\right)U^{MM}+\lambda_{t}U^{SQM/MM}$$

Here, $0\leq \lambda_{t}\leq 1$ is the coupling parameter which is incremented, e.g., linearly at each step of the switching simulation, according to

(5) $$\lambda_{t}=\lambda_{t}\left(i\right)=i/N_{switch},\;i=\;1,\;2,\ldots ,N_{switch}$$

In our protocols, $N_{switch}$ is, e.g., 2000 or 5000 steps, corresponding to a switching length of 2 or 5 ps with a timestep δt=1 fs, and we carry out two hundred of such switches (see below for details). Equation (5) describes the simplest switching protocol; in this work, we also test modified protocols where $\lambda_{t}$ is incremented at different speeds. The switching simulations are costly because the evaluation of the mixed potential energy function $U(\lambda_{t})$ requires the high-level energy function $U^{SQM/MM}$ and its gradient. However, since the switches are independent, they can be carried out in parallel. For each timestep at which $\lambda_{t}$ is incremented, the work is accumulated according to (cf. Ref. [44])

(6) $$W\left(t+\delta t\right)=W\left(t\right)+U\left(x_{t+\delta t},\lambda_{t+\delta t}\right)-U\left(x_{t+\delta t},\lambda_{t}\right)$$

One-sided methods, such as Jarzynski’s equation, are less efficient and reliable than the two-sided approaches [45]. The process outlined above to obtain $W^{MM\to SQM/MM}$ can be reversed, so one ends up with distributions $P^{MM\to SQM/MM}\left(W\right)$ of work values in the forward, and $P^{SQM/MM\to MM}\left(W\right)$ in the backward direction. Crooks showed that these two distributions are related according to [41]

(7) $$P^{MM\to SQM/MM}\left(W\right)=P^{SQM/MM\to MM}\left(-W\right)\exp\left[\beta \left(W-\Delta A^{MM\to SQM/MM}\right)\right]$$

from which one can deduce the free energy difference between the two states, i.e., between the two levels of theory. The notation in Equation (7) has been adapted to the present application. The practical use of Crooks’ equation is computationally costly, since one needs to generate equilibrium configurations at the high level of theory. As in the past, Crooks’ equation will therefore only be used to generate reference results to assess the convergence of free energy differences obtained using Jarzynski’s equation. In this work, Jarzynski’s and Crooks’ equations (or their use) are abbreviated as JAR and CRO, respectively.

The mixing of Hamiltonians as required by Equation (4) was realized with the MSCALE module of CHARMM [46], which makes it possible to combine energies and forces from different sources, including separate programs, in an almost arbitrary manner. The MSCALE functionality is coupled to one of CHARMM’s tools (PERT) to compute free energy differences [47]. Realizing that short/fast slow-growth thermodynamic integration calculations yield a non-equilibrium work rather than a converged free energy differences [48], one can exploit PERT’s slow-growth mode to carry out NEW switches. Using PERT, it is also possible to carry out stepwise linear switching protocols; see Section 4.3.2 below.

### 4.2. Choice of Model Systems

This study focuses on the optimization of the calculation of $\Delta A_{X\;solv}^{MM\to SQM/MM}$ (red arrow in Figure 1a); in other words, the free energy difference between a system in which all interactions are described using classical mechanics, and a hybrid system, in which a small region of interest is described using SQM, and the remainder described using MM. Examples are solute–solvent systems, in which the solute is modeled either via molecular mechanics or via quantum chemistry, whereas the solvent (water) is always treated classically. As outlined earlier, difficulties may arise from the differences in the charge distributions of the MM and SQM representations of the solute molecule X. To exclude other factors hindering convergence, suitable model systems should be relatively rigid and not have multiple conformational substates.

**Figure 10:**
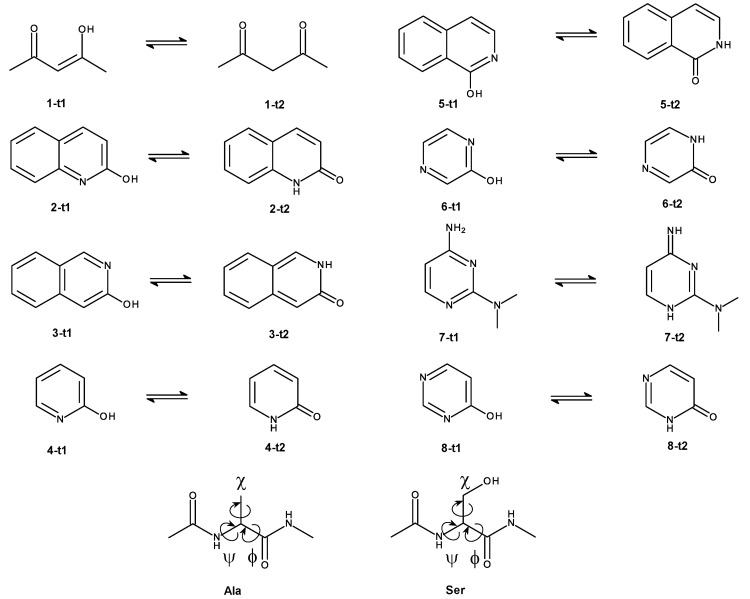
Model systems used in this work: eight tautomer pairs, seven taken from the SAMPL2 challenge [49], and one (**7-t1** ⇌ **7-t2**) from *Tautobase* [50]. In addition, we used the blocked amino acids **Ala** and **Ser** as model solutes.

Many tautomer pairs fulfil this requirement. Therefore, we picked seven such pairs from the tautomer part of the SAMPL2 challenge [49]. One additional pair was taken from *Tautobase* [50]. We also included N-acetyl-alanine-methylamide (**Ala**) and N-acetyl-serine-methylamide (**Ser**) as model solutes, since these were the two compounds for which we noticed slow convergences in Ref. [31]. All model compounds used in this work are shown in Figure 10.

The prediction of tautomer preferences in aqueous solution remains a challenging problem [51] and is out of the scope of this study. Here, we concentrate on finding efficient protocols to compute the correction $\Delta A_{X\;solv}^{MM\to SQM/MM}$, the red arrow in Figure 1. Since (S)QM/MM methods are likely necessary for answering questions involving tautomer preferences, the present work establishes the necessary methodology to enable such calculations efficiently.

### 4.3. Strategies to Improve the Convergences of NEW Simulations

#### 4.3.1. Hybrid Charge Intermediates

As outlined in the Introduction, cf. Figure 1b, we investigated whether the quantity of interest $\Delta A_{X\;solv}^{MM\to SQM/MM}$ can be computed more efficiently by inserting an intermediate state. We refer to it as “*MULL*”; the rationale for this label will become clear shortly. Thus, we have

(8) $$\Delta A_{X\;solv}^{MM\to SQM/MM}=\Delta A_{X\;solv}^{MM↔MULL}+\Delta A_{X\;solv}^{MULL\to SQM/MM}$$

This intermediate state should have a charge distribution resembling that of the SQM/MM end state. To be useful in practice, the free energy difference between the initial MM and the intermediate state $\Delta A_{X\;solv}^{MM↔MULL}$ must be easy (=fast) to compute. In practice, this means that the interactions at the intermediate state must also be classical. The obvious choice, therefore, is to use the force field of the initial MM state with suitably modified partial charges.

In all our methodological work to date [30,31,32,33,34,35,36], we used the SCC-DFTB SQM method [52,53] as the high level of theory. On the one hand, SCC-DFTB is expected to be sufficiently similar to ab initio QM methods, so that insights obtained will be transferable to full DFT QM methods. On the other hand, SCC-DFTB is fast enough to explicitly carry out simulations at the high level of theory, something which is not feasible for ab initio QM methods. The ability to carry out simulations at the SQM/MM level of theory makes it possible to obtain rigorous reference results using Crooks’ equation; cf. the previous section.

Since Mulliken charges and Mulliken charge analysis are at the core of the SCC-DFTB methodology [52,53], a logical choice for the intermediate state is the combination of the MM force field (see below for details) with fixed partial charges derived from the Mulliken charges used at the SCC-DFTB level of theory; hence, the superscript MULL in Figure 1b and Equation (8). In SCC-DFTB calculations, the converged Mulliken charges change at every simulation step; therefore, an averaging procedure is needed. There are several possibilities for how to obtain a representative sample of configurations for which Mulliken charges are computed. The following three approaches are tested in this study. (i) Clearly, the most exact method is to use simulations at the high level of theory (the solute treated using SCC-DFTB, surrounded by MM waters) to obtain configurations for which the Mulliken charges are calculated and then averaged. All simulations and results using this intermediate state are labeled MULL(solv*). Our goal, however, is to avoid simulations at the high level of theory as much as possible. Therefore, we also used configurations sampled at the MM level of theory to derive Mulliken charges for the intermediate representation. Specifically, we used (ii) the Mulliken charges obtained from snapshots of the MM gas phase simulation of the solute (labeled as *MULL(gas)*), or (iii) we used snapshots from the corresponding MM simulations in water to obtain Mulliken charges for the solute (labeled as *MULL(solv)*).

The three sets of MULL charges were obtained by averaging over configurations saved during equilibrium MD simulations, i.e., MULL(gas) charges were derived from the MM gas phase simulations, MULL(solv) from the MM simulations in solutions, and MULL(solv*) from the SQM/MM simulations in solution. These are the simulations needed to save restart files for the NEW switching simulations to the respective other levels of theory. As described below (cf. Section 4.4.1), we always carried out eight repetitions, i.e., eight MD simulations per state and system. From each of these, 25 configurations were taken and reevaluated at the SQM(/MM) level of theory, writing out the converged Mulliken charges. Thus, each MULL charge set was obtained by averaging over 200 configurations.

If one inserts one of the MULL intermediate states to compute $\Delta A^{MM\to SQM/MM}$ as described above, then one also must compute $\Delta A_{Xsolv}^{MM↔MULL}$ to close the thermodynamic cycle (cf. Figure 1b). Since the MM and the MULL states differ only in the partial atomic charges, we calculated $\Delta A_{Xsolv}^{MM↔MULL}$ using equilibrium free energy methods. Specifically, in addition to the end states, we used three equidistant intermediate states and calculated the free energy difference using Bennett’s acceptance ratio method (BAR) [42].

#### 4.3.2. Stepwise Linear Switching Protocols

All protocols that are employed to switch from MM to SQM/MM are quite rapid, so the system will not remain in equilibrium. In the context of slow-growth thermodynamic integration, this has been referred to as Hamiltonian lag, and has been shown to lead to poorly converged results [48,54,55]. Although JAR [40] makes it possible to obtain the equilibrium free energy difference for a process from multiple irreversible work values, the convergence of the results depends on the variance of the work values, which in turn depends on the deviation of the switching trajectories from the equilibrium conditions [44,56].

**Figure 11:**
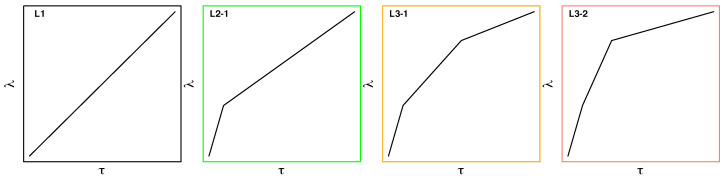
The standard linear (L1) and three modified switching protocols investigated. The total switching length τ was 2 ps in all four cases. The color code used for L2-1, L3-1, and L3-2 is also used in Results.

In addition to inserting an intermediate state with partial charges resembling the high level of theory, as just described, we explored the use of modified switching protocols. Results from solvation spectroscopy [38,39] indicate that the faster process of water reorientation in response to a change in the charge distribution of a solute is completed after approximately 0.5 ps. We therefore tested piecewise linear switching protocols consisting of two or three stages, referred to as L2-X and L3-X, respectively. The three protocols investigated in detail are shown in Figure 11, which also include the default linear protocol L1 of Equation (5) (leftmost plot). In all cases, the total switching length τ is 2000 fs. In L2-1, instead of incrementing $\lambda_{t}$ linearly, there are two stages: first, $\lambda_{t}$ is incremented from $\lambda_{t}=0.00\to 0.35$ in 200 fs, followed by slower linear switching from $\lambda_{t}=0.35\to 1.00$ over 1800 fs. The two L3-X protocols use three stages. In L3-1, $\lambda_{t}$ is switched from 0.0 to 0.35 to 0.8 to 1.0 over 200, 900, and 900 fs, respectively. Finally, L3-2 is very similar, but $\lambda_{t}$ is switched from 0.0 to 0.35 to 0.8 to 1.0 over 200, 400, and 1400 fs, respectively. In all modified protocols, $\lambda_{\tau }$ is initially changed more rapidly compared to L1, followed by a slower change in $\lambda_{\tau }$ during the remainder of the switch.

To verify the applicability and the performance of the modified compared to the standard linear switching protocol, systematic tests were carried out for five compounds (**1-t2**, **4-t2**, **5-t2**, **6-t2**, and **8-t1**). This selection was motivated by the results obtained with the linear protocol, and includes both “easy” and “difficult” solutes with respect to convergence (cf. Results, Section 2.2).

#### 4.3.3. Analyses Carried Out

The main focus of this study lies on computing $\Delta A_{Xsolv}^{MM\to SQM/MM}$ efficiently, and most of our results are concerned with the accuracy of JAR-based protocols compared to the reference results obtained with CRO. Additional analyses and characterizations were carried out to understand how solute–solvent interactions change and thus affect convergence when switching from an MM to an SQM representation of the solute.

##### Characterizing Charge Distributions

As described in Section 4.3.1, we use three intermediate charge distributions to facilitate the transformation from the low to the high level of theory. It is therefore of interest to quantify the differences between the charge sets used. In the following, the reference charge set is always the one that is derived from averaging over the Mulliken charges obtained from SQM/MM trajectories [MULL(solv*)]. To quantify the difference between two charge sets, we computed the root-mean-square deviation ${\mathrm{RMSD}}_{q}$, defined as

(9) $${\mathrm{RMSD}}_{q}=\sqrt{\frac{1}{N}\sum_{i=1}^{N}{\left(q_{i}^{method}-q_{i}^{MULL(solv*)}\right)}^{2}}$$

Here, *N* is the number of atoms of the molecule, the $q_{i}^{MULL(solv*)}$ charges serve as the reference, and the $q_{i}^{method}$ is one of the other charge sets used; i.e., *method* can be MM, MULL(gas), or MULL(solv).

Because all our solutes are neutral, the most important quantity affected by a change in partial charges is the dipole moment $\vec{\mu }$. To compare the dipole moments resulting from the various charge sets, we consider the “differential” dipole moments between two charge distributions of a solute, i.e.,

(10) $$\Delta \vec{\mu }=\sum_{i=1}^{N}\left(q_{i}^{method}-q_{i}^{MULL(solv*)}\right)\cdot \vec{r_{i}}$$

In practice, we mostly compare the length of this vector, i.e., $\left|\Delta \vec{\mu }\right|=\Delta \mu$.

Furthermore, the angle between the dipole moment vectors of two charge representations is of interest, which can be obtained in the usual way, is:

(11) $$\Theta_{\mathrm{SQM}}=\cos^{-1}\left(\frac{{\vec{\mu }}^{method}\cdot {\vec{\mu }}^{MULL(solv*)}}{|{\vec{\mu }}^{method}|\cdot |{\vec{\mu }}^{MULL(solv*)}|}\right)$$

Dipole moments were computed using the COOR DIPOle command of CHARMM (see https://academiccharmm.org/documentation/version/c47b1/corman (accessed on 29 April 2023)); if the difference between two charge sets is assigned as partial charges, one obtains the corresponding differential dipole moment.

##### Characterization of the First Solvation Shell

We analyzed all MM, MULL (gas), MULL (solv), MULL (solv*), and SQM/MM equilibrium trajectories to determine the average number of solvent water molecules in proximity to the solute. Specifically, a water molecule was considered to be in close contact if the distance between the water oxygen and a non-hydrogen atom of the solute was ≤3 Å. These analyses were carried out with the atom selection facility of CHARMM. (See https://academiccharmm.org/documentation/version/c47b1/select (accessed on 29 April 2023)).

##### Dynamics of Solvent Reorientation

To investigate the detailed dynamics of solvent reorientation upon changing the description of the solute from the MM to the SQM level of theory, we resort to ideas from computational spectroscopy, specifically the computation of the normalized Stokes shift S(t) from non-equilibrium MD simulations. S(t) is defined as [57,58]:

(12) $$S\left(t\right)=\frac{\nu (t)-\nu (\infty )}{\nu (0)-\nu (\infty )}\approx \frac{\bar{\Delta U(t)}-\bar{\Delta U(\infty )}}{\bar{\Delta U(0)}-\bar{\Delta U(\infty )}}$$

where ν(t) is the time-dependent frequency of the emitted fluorescence light of the probe molecule’s chromophore. In computation, one assumes that changes in interactions between the solute and the solvent leading to ν(t) are purely electrostatic in nature. Thus, one takes equilibrated configurations from solute–solvent simulations, changes the partial charges from those describing the ground state to those describing the excited state, restarts the simulations, and monitors the difference in electrostatic solute–solvent interactions between the ground ($U^{0}$) and the excited state ($U^{*}$), $\Delta U\left(t\right)=U^{*}\left(t\right)-U^{0}\left(t\right)$ as a function of time. Just as the measured fluorescence signal is an average of the emissions from all the chromophores present in solution, one averages over at least a few hundred simulations; hence the bar in $\bar{\Delta U(t)}$.

In the context of our work, the low- and high-level representations of the solute (region of interest) play the roles of the ground and excited states in solvation spectroscopy. In both cases, the solvent water has to reorient itself following a change in the charge distribution of the solute. Therefore, we adopted the computational approach to compute S(t) [57,58] as follows. We used starting configurations equilibrated either at (i) the MM level of theory or (ii) the MULL(solv) intermediate state, and restarted simulations with the full SQM/MM description of interactions. For each system studied (**4-t2**, **5-t2**, **6-t2** and **8-t2**), we computed 800 simulations of 10 ps length; based on the experimental findings, it is reasonable to expect that solvent reorientation is completed after this time [38,39]. At each timestep, we saved $\Delta U\left(t\right)=U^{SQM/MM}\left(t\right)-U^{method}\left(t\right)$, where *method* was either MM or MULL(solv). This task was facilitated by a locally modified version of CHARMM, but could equally well be accomplished through the post-processing of trajectories saved during the simulations. The 800 ΔU(t) time series were then averaged, resulting in the averaged time series $\bar{\Delta U(t)}$. Next, we estimated $\bar{\Delta U(\infty )}$, i.e., the limit of t→∞, by averaging (again) over the last 2000 entries of $\bar{\Delta U(t)}$ (8ps≤t≤10ps).

Equation (12) is the definition of the normalized Stokes shift, whereas in the present context omitting the normalization, it turned out to be advantageous. Thus, we operate with the averaged time series,

(13) $$\bar{\Delta \Delta U(t)}=\bar{\Delta U(t)}-\bar{\Delta U(\infty )},$$

which essentially is an unnormalized Stokes shift. To interpret it more easily, $\bar{\Delta \Delta U(t)}$ was fitted to a mono-exponential target function

(14) $$\bar{\Delta \Delta U(t)}\propto \Delta U_{0}\cdot e^{-t/\tau }$$

where $\Delta U_{0}$ is the prefactor of the exponential decay at t=0, *t* is the time in fs, and τ the relaxation time constant in fs. As described in the Introduction, it is known experimentally that the orientation of solvent water occurs on two timescales [38,39]. Because any effects on the convergence of JAR calculations will result from the slower process, we intentionally always discarded the first 0.5 ps when carrying out the fit. Thus, the τ found using the fitting procedure should correspond to the slower reorientation process.

### 4.4. Overview of Simulations Carried Out

All simulations were carried out with CHARMM (developmental versions c44a2 and c47a1) [47]. The calculation of $\Delta A_{Xsolv}^{MM\to SQM/MM}$ requires equilibrium simulations to generate the configurations from which the NEW switching simulations are started. As described above, in addition to switching directly from the MM to the SQM/MM level of theory, we also inserted three force field-based intermediate states with different atomic partial charges. For each of these states, we also had to carry out equilibrium simulations, followed by NEW switches to the SQM/MM level of interest. The NEW switching simulations themselves were carried out either in a strictly linear fashion (protocol L1 in Figure 11), or with a two- or three-staged linear protocol. To make sure that any convergence problems observed did not arise without solvent, we also carried out gas phase simulations for all compounds shown in Figure 10 using the optimized protocol of Ref. [36].

#### 4.4.1. Simulation Details

##### Preparation and Initial Equilibration

For each molecule shown in Figure 10, starting coordinates were generated using CHARMM-GUI [59,60]. Missing force field parameters were generated using CGenFF 2.4.0 [61,62,63], as invoked by CHARMM-GUI. The solute–solvent systems were set up by placing each molecule into a cubic box of water molecules with an initial side-length of 26 Å containing 572 (CHARMM-modified) TIP3 waters [64]. Any water molecule overlapping with a solute atom was deleted. Each system was then equilibrated as follows: 100 steps of steepest descent minimization were followed by a constant pressure/temperature (CPT) simulation of 100 ps length with a timestep of 1 fs, applying the Langevin piston barostat [65] (mass of the pressure piston: 400 Da, Langevin piston collision frequency: 20 ps^−1^, Langevin piston bath temperature: 300 K). The final 20 ps of these equilibration runs were used to determine the average box size. In Table S1 of the SI, we list the size of the simulation box determined in this manner, as well as the number of water molecules present, for each of the compounds studied.

##### Force Field-Based Equilibrium Simulations

Starting from the initial solute coordinates and the equilibrated solute–solvent systems, eight Langevin dynamics (LD) simulations (timestep 1 fs, friction coefficient 5 ps^−1^) were carried out in the gas phase and in solution, respectively. Each of these simulations was initialized with different random velocities drawn from a Maxwell-Boltzmann distribution at 300 K. In each gas phase run, 5 ns of simulation time were discarded as equilibration, followed by 10 ns of production, during which the restart files were saved every 1000th step. In the analogous solution simulations at constant volume, 0.5 ns were discarded as equilibration. During the subsequent 1 ns production phase, restart files were saved every 1000th step. Thus, during the cumulative simulation length of 8 ns (solution)/80 ns (gas phase), 8000 (solution)/80,000 (gas phase) restart files were saved. These served as the pool of configurations sampled in the canonical ensemble, from which non-equilibrium switching simulations to the high (SQM(/MM)) level of theory were started.

The solute molecules were always fully flexible; the TIP3 waters were held rigid using SHAKE [66]. In the gas phase, the nonbonded interactions were not truncated (“infinite” cutoff radius). In the solution simulations, Lennard-Jones interactions were smoothly switched off between 10 and 12 Å, and electrostatic interactions were computed with the particle-mesh-Ewald method [67] (κ=0.34 Å^−1^, spline interpolation to order 6, FFT grid size of 32).

The protocol just described was used both for the simulations at the MM level of theory, i.e., using the CGenFF force field, and for all simulations with a hybrid intermediate representation (force field with Mulliken-derived partial charges).

##### SQM(/MM) Equilibrium Simulations

To calculate the reference values via Crook’s equation, we also carried out simulations at the SQM(/MM) level of theory. Specifically, the solute was treated with the self-consistent-charge density-functional tight-binding (SCC-DFTB) method, as implemented in CHARMM [68], using the 3ob-3-1 [69,70,71,72] parameter set (https://www.dftb.org/parameters/download/3ob/3ob-3-1-cc/(accessed on 29 April 2023)). Water molecules were always treated classically. Analogous to the MM case just described, eight LD simulations started with different random initial velocities were carried out in the gas phase and in solution. The simulation length in all cases was 1 ns (1 million steps with a timestep of 1 fs). Restart files were written every 1000th step in solution and every 100th step in the gas phase, thus resulting in a total of 800 (solution)/8000 (gas phase) restart files generated during a cumulative simulation length of 8 ns. Nonbonded interactions were treated identically, as in the MM case.

#### 4.4.2. Non-Equilibrium Work Simulations

From each set of restart files written during the equilibrium simulations at the MM, MULL, and SQM(/MM) levels of theory, every 40th was selected as the starting point for a NEW switch to the respective other level of theory. Thus, 200 switches per molecule were carried out to compute the free energy difference between two levels of theory. In all switching simulations, we used a timestep of 1 fs. Unless otherwise noted, the switching length was 2000 fs (2000 steps), both for linear and stepwise linear switching paths.

#### 4.4.3. Calculation of $\Delta A_{Xsolv}^{\mathrm{MM}\;↔\;\mathrm{MULL}}$:

As described in Section 4.3.1, to close the thermodynamic cycle, one needs to compute $\Delta A_{Xsolv}^{\mathrm{MM}\;↔\;\mathrm{MULL}}$. Since the only difference between the MM and MULL descriptions of a system are the partial charges of the solute, $\left\{q_{i}\right\}$, intermediate states can be set up easily according to $q_{i}\left(\lambda \right)=\left(1-\lambda \right)q_{i}^{MM}+\lambda q_{i}^{MULL}$. To ensure an overlap between neighboring states, we used three intermediate states (λ=0.25,0.5,0.75) in addition to the MM (λ=0) and MULL (λ=1) end states. All simulation settings were the same as described in the force field-based equilibrium simulations; the production length per state was 1 ns. At each λ state, 1000 coordinate sets were saved and the energies were reevaluated at the neighboring states. The free energy difference between the neighboring states was computed with BAR; these were summed up to give $\Delta A_{Xsolv}^{\mathrm{MM}\;↔\;\mathrm{MULL}}$. All calculations were repeated eight times, starting from initial random velocities. The standard deviation obtained from these eight repetitions were used to estimate the standard error via Gaussian error propagation. All MM calculations were carried out with the OpenMM [73] GPU acceleration available in CHARMM (https://academiccharmm.org/documentation/version/c47b1/openmm(accessed on 29 April 2023)).

## 5. Conclusions

In summary, the reliable calculation of free energy differences between the MM and SQM/MM levels of theory in aqueous solution require either switching lengths of at least 5 ps, or the use of an appropriate intermediate charge state. In terms of aiding convergence, the use of intermediate states is not new. For example, the internal degrees of freedom of a molecule or region that should be described at two levels of theory can be made more similar to the target high-level representation via force matching [29,33,74]. Thus, the use of intermediate charge states plays the same role for electrostatic interactions between the core region (the region changed from e.g., MM to SQM) and the environment (the region always described at the low level of theory), as force-matched parameters play for the bonded energy terms of the core region. Since intermediate charge states can be rationally designed without too much computational effort, the poor convergence of MM→SQM/MM simulations due to slow solvent reorientation can be easily circumvented.

## Figures and Tables

**Table 1:** MAD (mean absolute deviation) for the results shown in Figure 3, as well as the spread of δΔA (respectively, the smallest and largest absolute deviations).

Pathway	MAD [kcal/mol]	Spread MAD [kcal/mol]	
		Min	Max
MM	0.29	0.01	1.80
MULL(gas)	0.12	0.01	0.49
MULL(solv)	0.09	0.01	0.28
MULL(solv*)	0.07	0.01	0.20

**Table 2:** MAD (Mean Absolute Deviation) over all compounds for ${\mathrm{RMSD}}_{q}$, the magnitude of the differential dipole moment Δμ, and the angle $\Theta_{\mathrm{SQM}}$ between the dipole moment vector of the respective charge distribution and that of the MULL(solv*) charge distribution.

Pathway	MAD RMSD${}_{q}\left[e\right]$	MAD Δμ [D]	MAD $\theta_{SQM}$[°]
MM	0.14	3.38	29.8
MULL(gas)	0.06	2.44	7.6
MULL(solv)	0.04	1.81	16.0

## Data Availability

Any additional data not available in the main manuscript or the SI are available on request from the corresponding authors.

## Supplementary Materials

The following supporting information can be downloaded at: https://www.mdpi.com/article/10.3390/molecules28104006/s1, Figure S1: δΔA including error bars; Figure S2: δΔA, all points; Figure S3: δΔA, all points, including error bars; Figure S4: RMSD[q]; Figure S5: Δμ[D]; Figure S6: $\theta_{SQM}{[}^{\circ }]$; Figure S7: 3-t2 charge analysis; Figure S8: 4-t2 charge analysis; Figure S9: 8-t2 charge analysis; Figure S10: 4-t2 TIP3P relaxation; Figure S11: 6-t2 TIP3P relaxation; Figure S12: 8-t2 TIP3P relaxation; Table S1: Additional information on model compounds; Table S2: Summary table of all δΔA results in solution; Table S3: Detailed results for $N_{switch}$ = 2000 fs $N_{replicate}$ = 200 $\Delta A_{Xsolv}^{MM\to SQM}$ in solution; Table S4: Detailed results for $N_{switch}$ = 2000 fs $N_{replicate}$ = 200 $\Delta A_{gas}^{MM\to SQM}$; Table S5: Detailed results for $N_{switch}$ = 5000 fs $N_{replicate}$ = 200 $\Delta A_{Xsolv}^{MM\to SQM}$ in solution; Table S6: Detailed results for $N_{switch}$ = 2000 fs $N_{replicate}$ = 200 $\Delta A_{Xsolv}^{MULL(gas)\to SQM}$ in solution; Table S7: Detailed results for energy correction term via FEP $\Delta A_{Xsolv}^{MULL(gas)\to SQM}$ in solution; Table S8: Detailed results for $N_{switch}$ = 2000 fs $N_{replicate}$ = 200 $\Delta A_{Xsolv}^{MULL(solv)\to SQM}$ in solution; Table S9: Detailed results for energy correction term via FEP $\Delta A_{Xsolv}^{MULL(solv)\to SQM}$ in solution; Table S10: Detailed results for $N_{switch}$ = 2000 fs $N_{replicate}$ = 200 $\Delta A_{Xsolv}^{MULL(solv*)\to SQM}$ in solution; Table S11: Detailed results for energy correction term via FEP $\Delta A_{Xsolv}^{MULL(solv*)\to SQM}$ in solution; Table S12: Mulliken Charge Analysis; Table S13: TIP3P Water Relaxation. Refs. [75,76] are cited in the Supplementary Materials.

## Abbreviations

The following abbreviations are used in this manuscript:

MD	Molecular Dynamics
MM	Molecular Mechanics
(S)QM	(Semi-empirical-)Quantum Mechanics
(S)QM/MM	(Semi-empirical-)Quantum Mechanical/ Molecular Mechanical hybrid methods
FES	Free energy simulation
FEP	Free energy perturbation
TI	Thermodynamic integration
BAR	Bennett’s acceptance ratio
NEW	Non-equilibrium work methods
JAR	Jarzynski’s Equation
CRO	Crook’s Equation
