# Supplementary material for: Exploring Routes to Enhance the Calculation of Free Energy Differences via Non-Equilibrium Work SQM/MM Switching Simulations Using Hybrid Charge Intermediates between MM and SQM Levels of Theory or Non-Linear Switching Schemes
Source: Molecules. 2023 May 10;28(10):4006. doi: 10.3390/molecules28104006 (PMC10222338; doi:10.3390/molecules28104006)
Supplement: Supplementary file 1 [file molecules-28-04006-s001.zip › molecules-2338263-supplementary.pdf]

List of Tables

S1. Additional information on model compounds . . . 2
S2. Summary table of all $\delta\Delta A$ results in solution . . . 11
S3. Detailed gas phase results for N_{switch}=2000 fs $N_{replicate}$=200 $\Delta A_{gas}^{MM\rightarrow SQM}$ . . . 11
S4. Detailed results for N_{switch}=2000 fs $N_{replicate}$=200 $\Delta A_{solv}^{MM\rightarrow SQM}$ in solution . . . 12
S5. Detailed results for N_{switch}=5000 fs $N_{replicate}$=200 $\Delta A_{solv}^{MM\rightarrow SQM}$ in solution . . . 12
S6. Detailed results for N_{switch}=2000 fs $N_{replicate}$=200 $\Delta A_{solv}^{MULL(gas)\rightarrow SQM}$ in solution . . . 13
S7. Detailed results for energy correction term via FEP $\Delta A_{solv}^{MM\leftrightarrow MULL(gas)}$ in solution . . . 13
S8. Detailed results for N_{switch}=2000 fs $N_{replicate}$=200 $\Delta A_{solv}^{MULL(solv)\rightarrow SQM}$ in solution . . . 14
S9. Detailed results for energy correction term via FEP $\Delta A_{solv}^{MM\leftrightarrow MULL(solv)}$ in solution . . . 14
S10. Detailed results for N_{switch}=2000 fs $N_{replicate}$=200 $\Delta A_{solv}^{MULL(solv*)\rightarrow SQM}$ in solution . . . 15
S11. Detailed results for energy correction term via FEP $\Delta A_{solv}^{MM\leftrightarrow MULL(solv*)}$ in solution . . . 15
S12. Mulliken Charge Analysis . . . 16
S13. TIP3P Water Relaxation . . . 17

List of Figures

S1. $\delta\Delta A$ including error bars . . . 4
S2. $\delta\Delta A$ all points . . . 4
S3. $\delta\Delta A$ all points including error bars . . . 5
S4. RMSD[q] . . . 5
S5. $\Delta\mu$[D] . . . 6
S6. $\theta_{SQM}[^\circ]$ . . . 6
S7. 3-t2 Charge analysis . . . 7
S8. 4-t2 Charge analysis . . . 7
S9. 5-t2 Charge analysis . . . 7
S10. 6-t2 Charge analysis . . . 8
S11. 8-t2 Charge analysis . . . 8
S12. 4-t2 TIP3P Relaxation . . . 9
S13. 6-t2 TIP3P Relaxation . . . 9
S14. 8-t2 TIP3P Relaxation . . . 10

Table S1. Compound label, total number of atoms (N_{total}), total number of heavy atoms (N_{heavy}), number of TIP3P waters (N_{Waters}), box length and free energy offset for each compound investigated. This offset has to be subtracted (added) to the respective $\Delta A^{MM \rightarrow SQM}$ ($\Delta A^{SQM \rightarrow MM}$) listed in Tables S3–S11 below.

Compound	N_{tot}/N_{heavy}	N_{Waters}	Box length [Å]	Offset[kcal/mol]
**1-t1**	15/7	560	25.588	11000
**1-t2**	15/7	561	25.802	11000
**2-t1**	18/11	559	25.630	14000
**2-t2**	18/11	559	25.576	14000
**3-t1**	18/11	559	25.595	14000
**3-t2**	18/11	559	25.596	14000
**4-t1**	12/7	561	25.589	10000
**4-t2**	12/7	561	25.573	10000
**5-t1**	18/11	560	25.622	14000
**5-t2**	18/11	559	25.582	14000
**6-t1**	11/7	560	25.562	10000
**6-t2**	11/7	561	25.557	10000
**7-t1**	20/10	560	25.612	14000
**7-t2**	20/10	560	25.581	14000
**8-t1**	11/7	562	25.559	10000
**8-t2**	11/7	561	25.556	10000
**Ala**	22/10	561	25.601	16000
**Ser**	23/11	561	25.622	18000

Overview of detailed results tables:

Tables S2–S11 summarize all raw data used to generate the tables and figures shown in the main manuscript. In this study, the free energy differences $\Delta A^{MM\leftrightarrow SQM}$ obtained by CRO (Crook's equation)[1] with switching lengths of 5 ps were considered as the reference results and are referred to as CRO_{Ref}. The deviations $\delta\Delta A$ from CRO_{Ref} for each of the methods studied (cf. Eq. 13 of the main manuscript) are listed in Table S2. Figures 4 and 5 of the main manuscript are based on the values in Table S2. Table S2 also provides the standard error, obtained by Gaussian error propagation of the individual results.

Tables S3–S6, S8 and S10 contain the detailed results obtained with Jarzynski's equation (JAR)[2] in the forward (MM→SQM/MM) and backward (SQM/MM→MM) direction, as well as the corresponding CRO result using the *same* switching length as for the JAR calculations. In addition to various convergence criteria, see below, we also report the difference between the JAR(fw) and the CRO result with this switching length (rightmost column in Tables S3 - S6, S8 and S10). Since in Table S4 we report results obtained with 2 ps switching length, the difference JAR(fw) − CRO reported there is slightly different from the corresponding $\delta\Delta A$ entry in Table S2 (leftmost column). By contrast, JAR(fw) − CRO in Table S5, $N_{switch} = 5000$ fs, is identical to the corresponding $\delta\Delta A$ entry in Table S2. Tables S6, S8 and S10 summarize JAR and CRO results for the three sets of simulations using an intermediate MM state with modified partial charges. To obtain the full free energy difference $\Delta A^{MM\leftrightarrow SQM}$, a correction step, the free energy difference between the force field description of the solute and the description with modified charges, needs to be computed; these results are summarized in Tables S7, S9 and S11. The overall deviation $\delta\Delta A$ of this two-step process from CRO_{Ref} are also reported in the three right-most columns in Table S2.

Convergence criteria reported in Tables S3–S6, S8 and S10:

In line with earlier work,[3] we computed several convergence criteria for each free energy difference $\Delta A^{MM\rightarrow SQM}$. As described in the main manuscript, raw data for each ΔA was generated using eight independent series of calculations. We consider each set of raw data as a block and calculated ΔA_i for each of these blocks. Thus, we can compute a "hysteresis" Hyst(eresis) $= \Delta A - 1/8 \sum_{i=1}^{8} \Delta A_i$; cf. Ref. 4. Further, we report the one-sided Π values for the forward and backward free energy differences.[5] Π values > 0.5 indicate that the W distribution is based on sufficient and unbiased sampling. We report two variants for Π, one evaluated with the initial guess for ΔA calculated by CRO (Π_{CRO}),the other by JAR (Π_{JAR}).

Additional convergence criteria reported in Tables S7, S9 and S11:

In the present work three intermediate MM states were used, which differed from the force field description by the partial charges used for the solute. To complete the thermodynamic cycle, one has to compute the respective free energy difference between the force field (MM) state and the Mulliken charge intermediate state (MULL). Here, this correction $\Delta A^{MM\leftrightarrow MULL}$ was calculated by (two-sided) free energy perturbation, i.e., Bennett's acceptance ratio method (BAR, cf. Sect. *Hybrid charge intermediates* of the main manuscript). The standard deviation $\sigma\Delta A$ was estimated by block averaging. In addition, we report the error for closing the cycle shown in Fig. 1b of the main manuscript, i.e., the deviation from ideality,

$$\Delta A^{MM\leftrightarrow MULL} + \Delta A^{MULL\rightarrow SQM} - \Delta A^{MM\leftrightarrow SQM} = 0,$$

using the 5 ps CRO results as the reference value. This cycle closure is labelled as BAR+JAR(fw)-CRO_{Ref} in the tables.

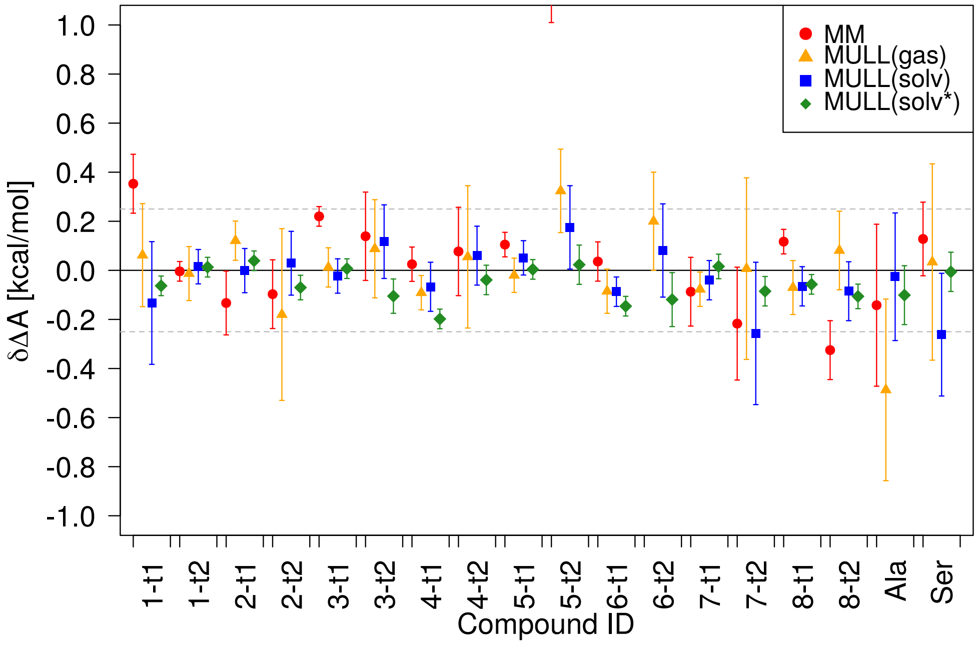

Figure S1. $\delta\Delta A$ calculated as the difference between one-sided method JAR(fw) and two-sided method CRO_{Ref} for switching forward($MM^* \rightarrow SQM$) from standard MM charges (MM), Hybrid charge intermediate based on MM gas phase simulation (MULL(gas)), Hybrid charge intermediate based on MM explicit solvent simulation (MULL(solv)) and Hybrid charge intermediate based on SQM/MM explicit solvent simulation (MULL(solv*)) including standard error.

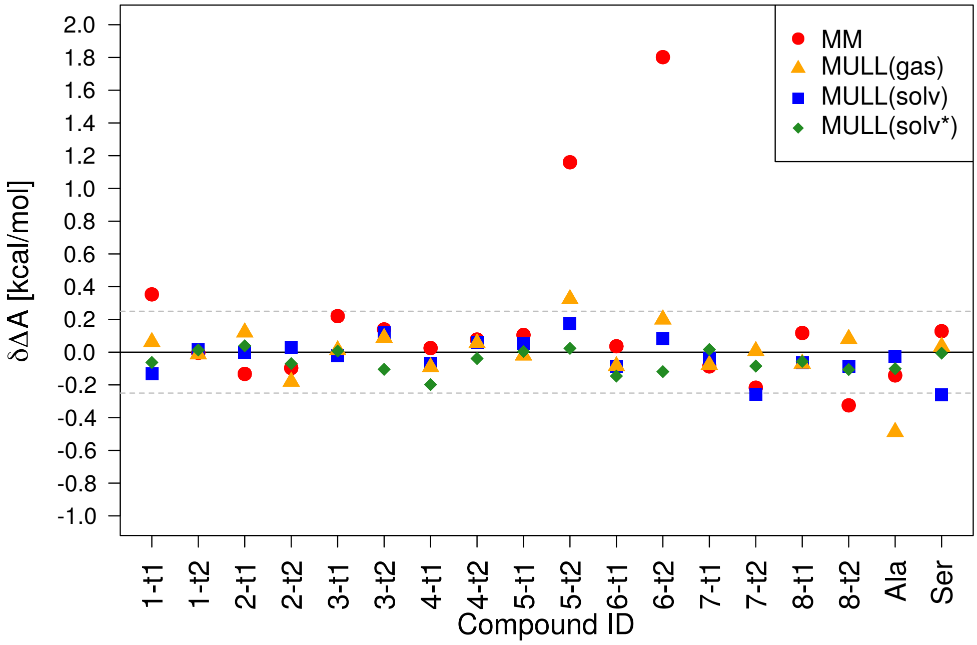

Figure S2. $\delta\Delta A$ calculated as the difference between one-sided method JAR(fw) and two-sided method CRO_{Ref} for switching forward($MM^* \rightarrow SQM$) from standard MM charges (MM), Hybrid charge intermediate based on MM gas phase simulation (MULL(gas)), Hybrid charge intermediate based on MM explicit solvent simulation (MULL(solv)) and Hybrid charge intermediate based on SQM/MM explicit solvent simulation (MULL(solv*)).

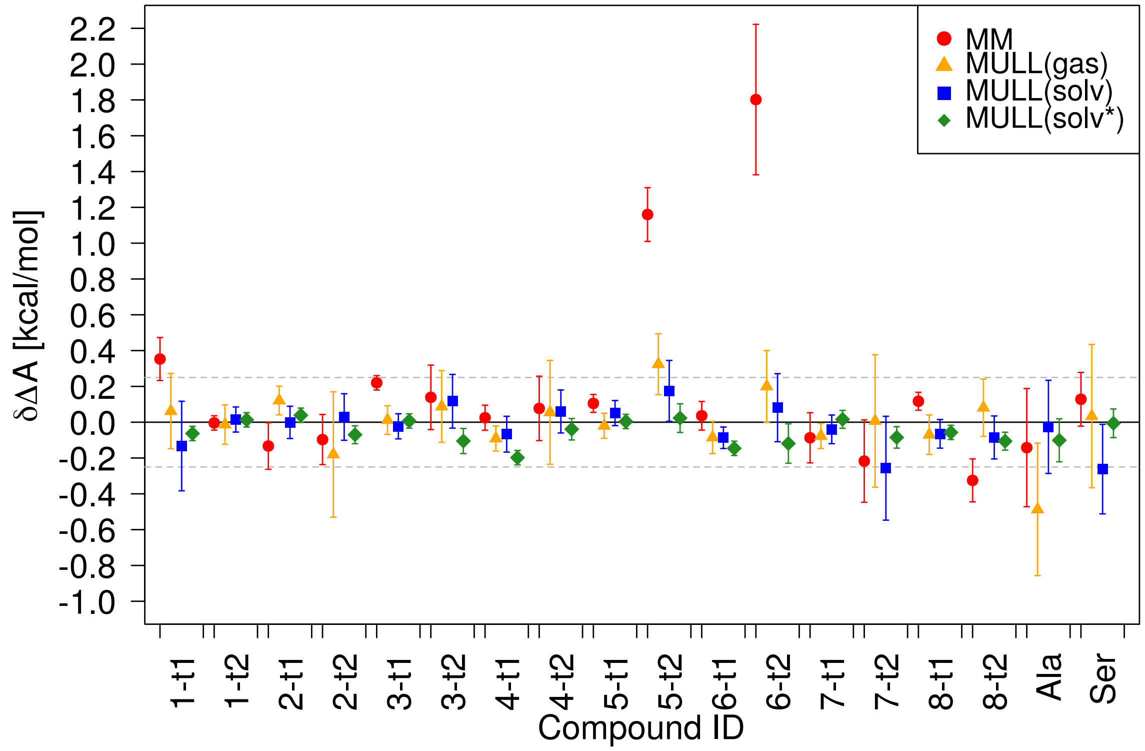

Figure S3. $\delta\Delta A$ calculated as the difference between one-sided method JAR(fw) and two-sided method CRO_{Ref} for switching forward($MM^* \rightarrow SQM$) from standard MM charges (MM), Hybrid charge intermediate based on MM gas phase simulation (MULL(gas)), Hybrid charge intermediate based on MM explicit solvent simulation (MULL(solv)) and Hybrid charge intermediate based on SQM/MM explicit solvent simulation (MULL(solv*)) including standard error.

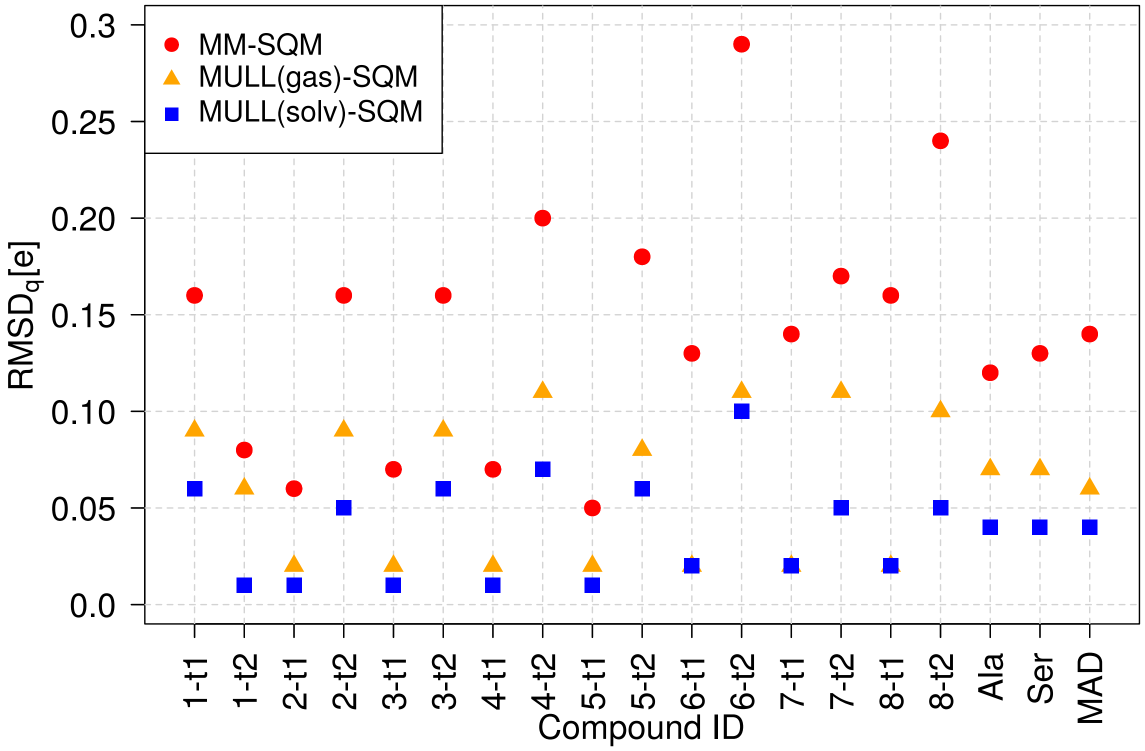

Figure S4. Root Mean square deviation of partial charge for all compounds; the MAD is plotted as well.

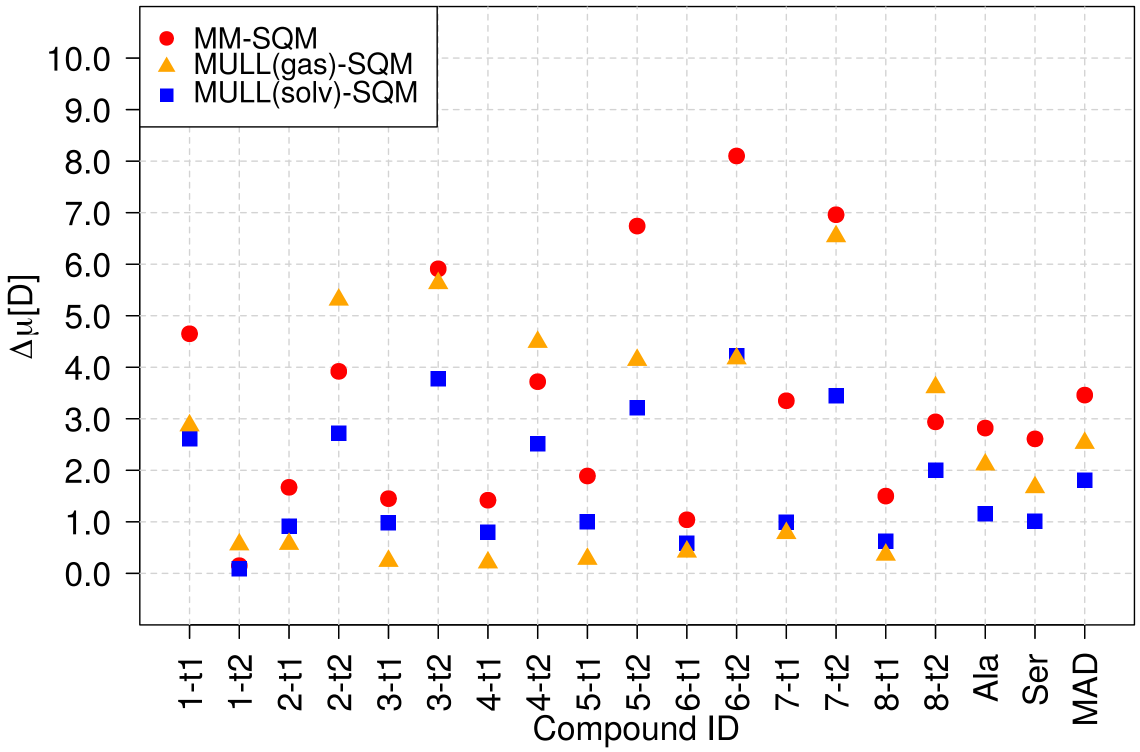

Figure S5. Differential dipole moment for all compounds, including MAD.

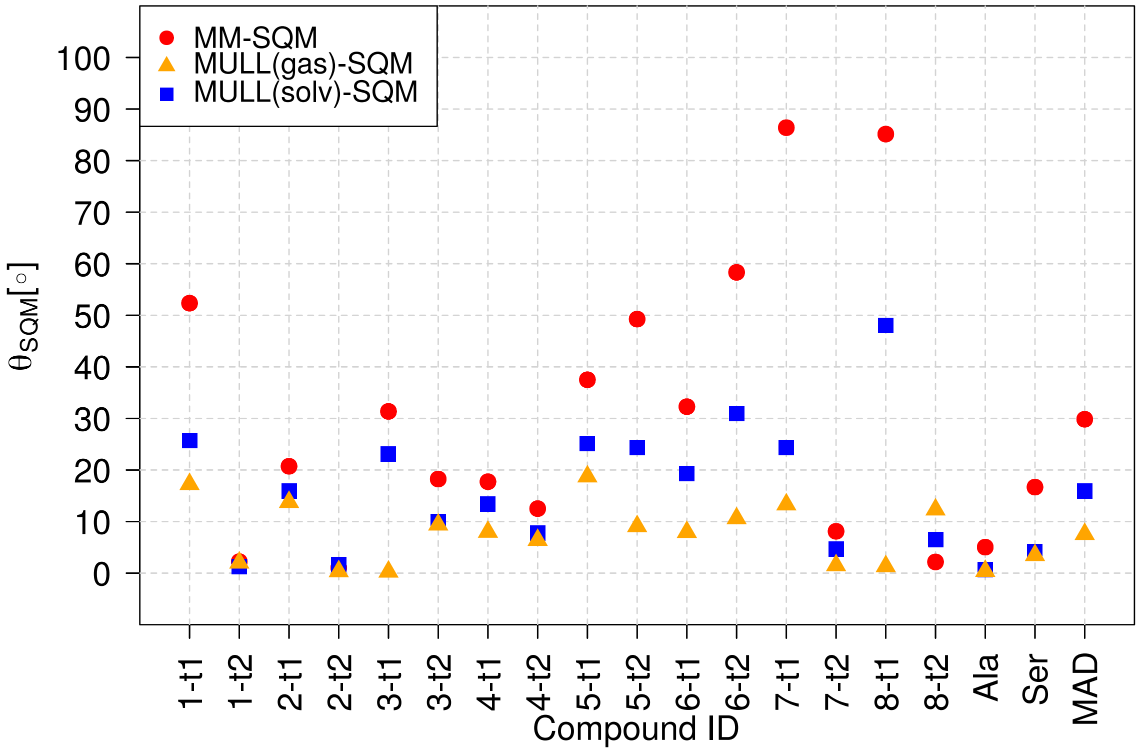

Figure S6. Angle between dipole moments for all compounds to SQM dipole moment, including MAD.

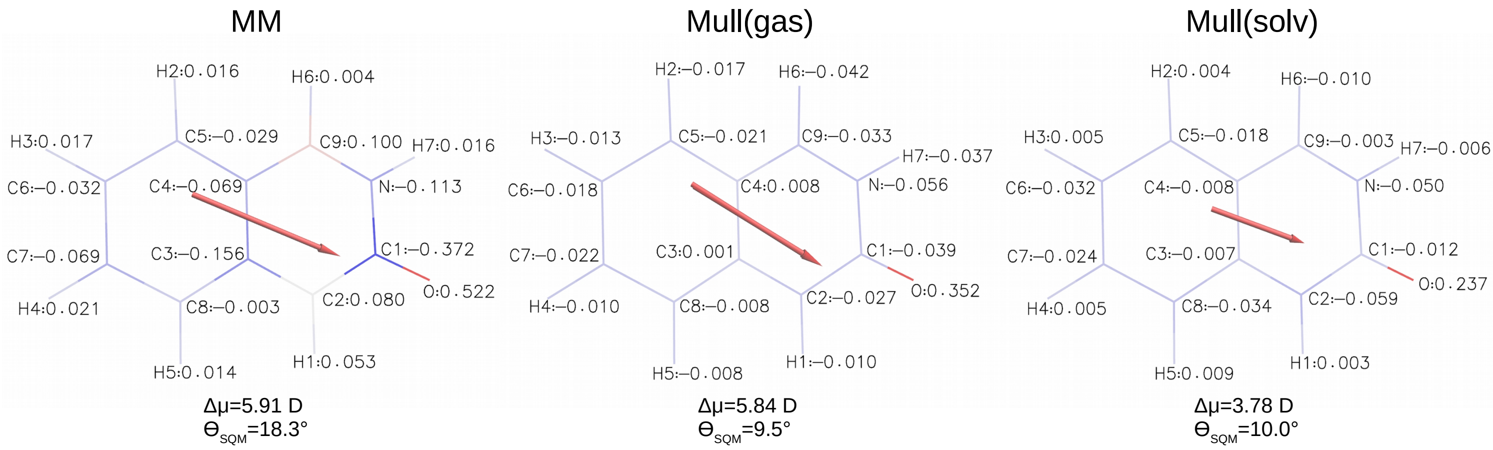

Figure S7. Differential dipole moments and atomic charges for **3-t2** including angle to SQM.

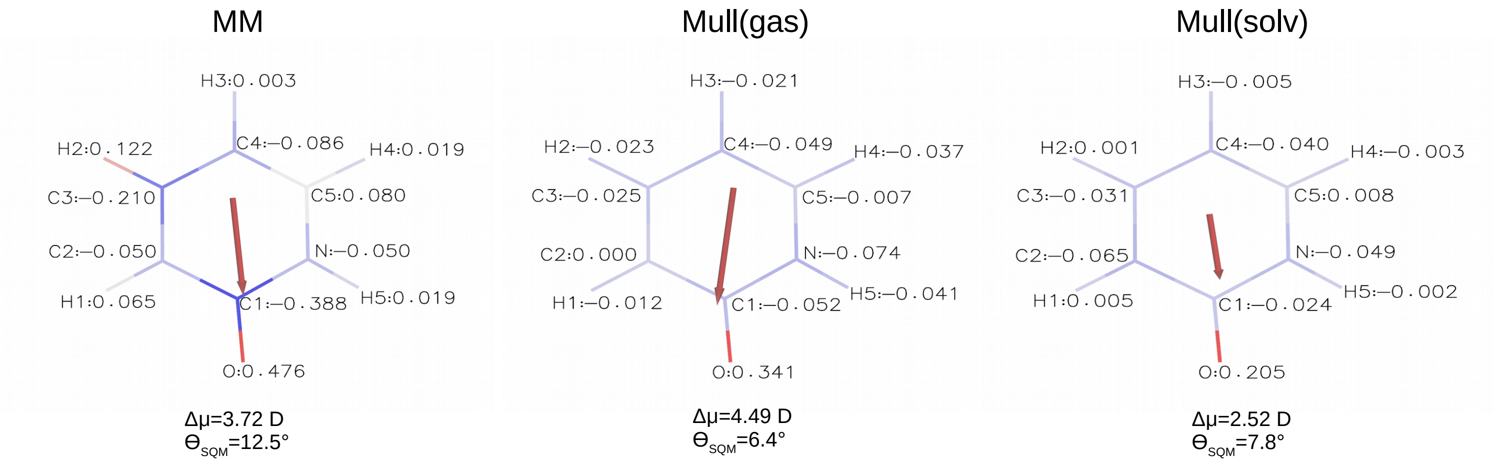

Figure S8. Differential dipole moments and atomic charges for **4-t2** including angle to SQM.

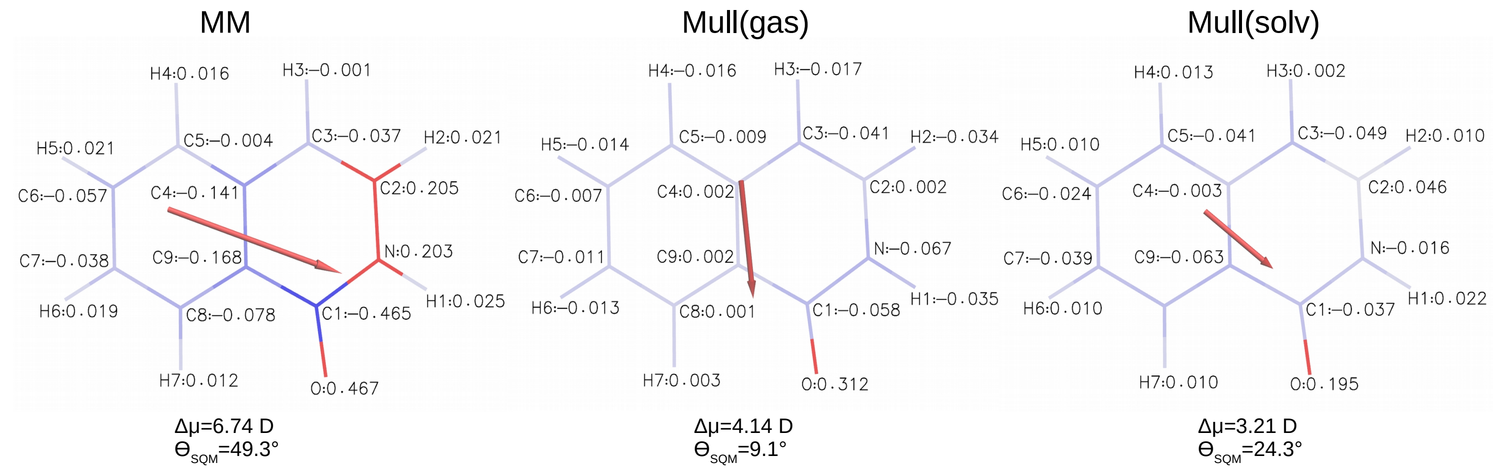

Figure S9. Differential dipole moments and atomic charges for **5-t2** including angle to SQM.

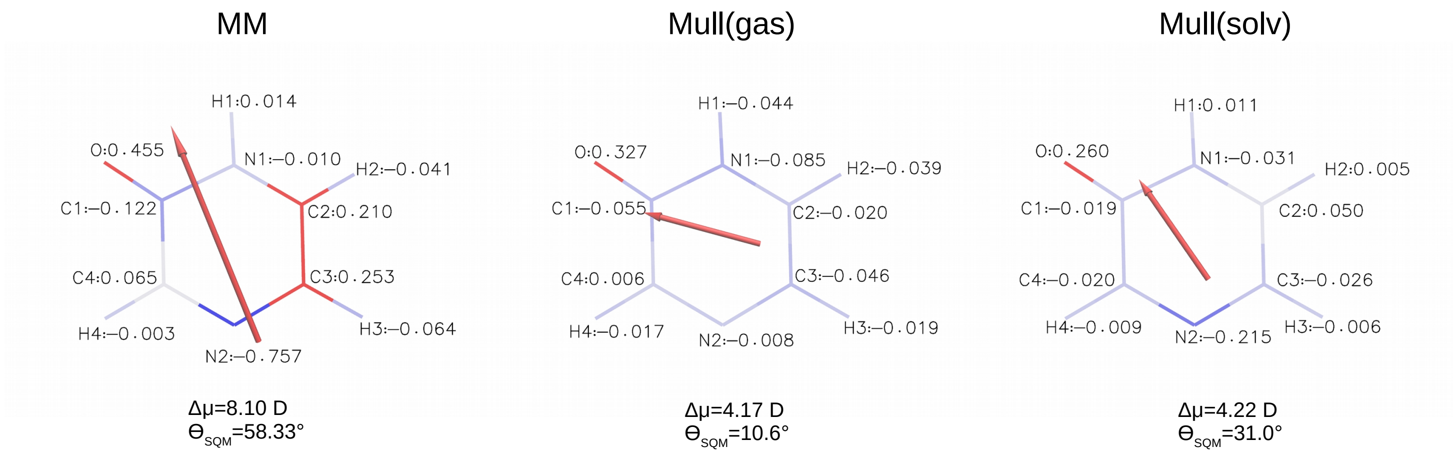

Figure S10. Differential dipole moments and atomic charges for **6-t2** including angle to SQM.

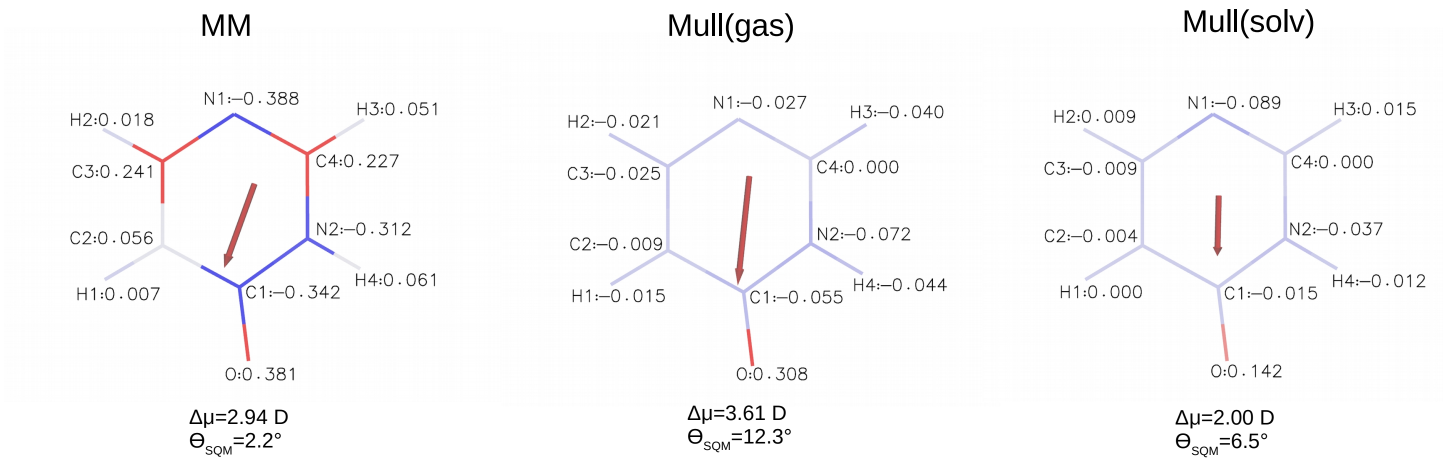

Figure S11. Differential dipole moments and atomic charges for **8-t2** including angle to SQM.

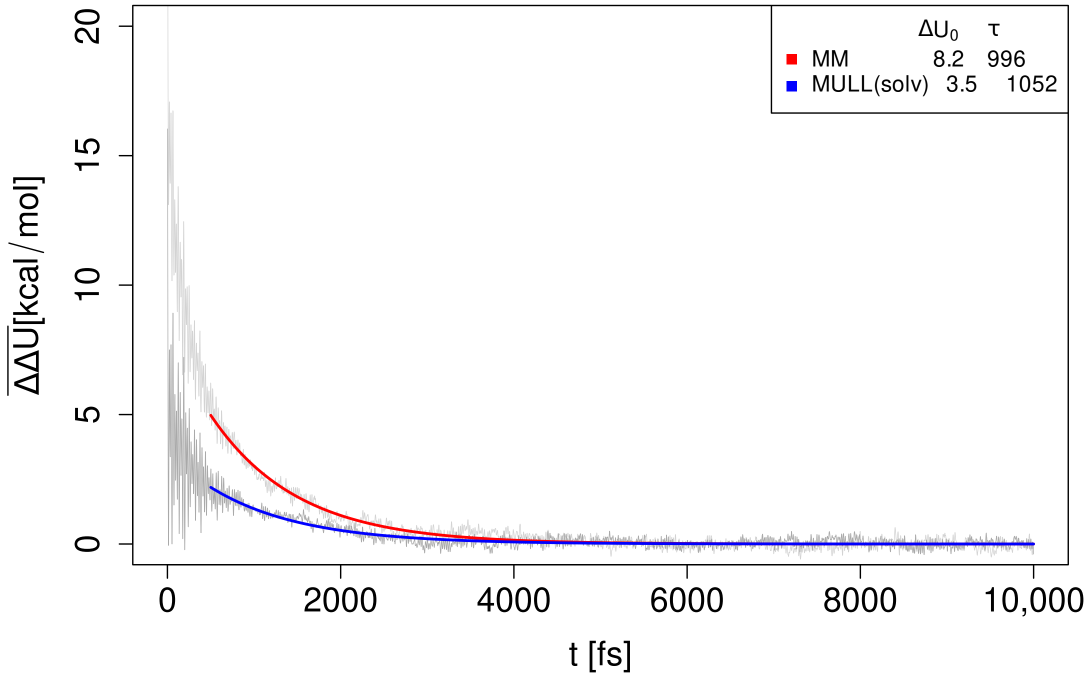

Figure S12. Relaxation of TIP3P Water for **4-t2** when switching from MM and MULL(solv) to SQM.

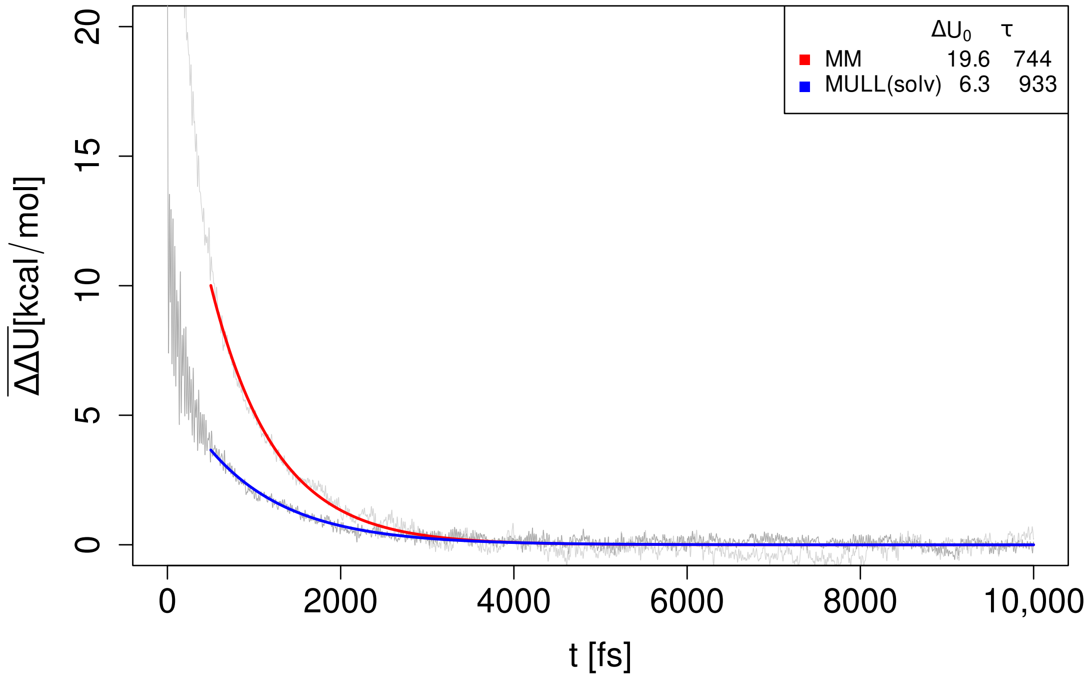

Figure S13. Relaxation of TIP3P Water for **6-t2** when switching from MM and MULL(solv) to SQM.

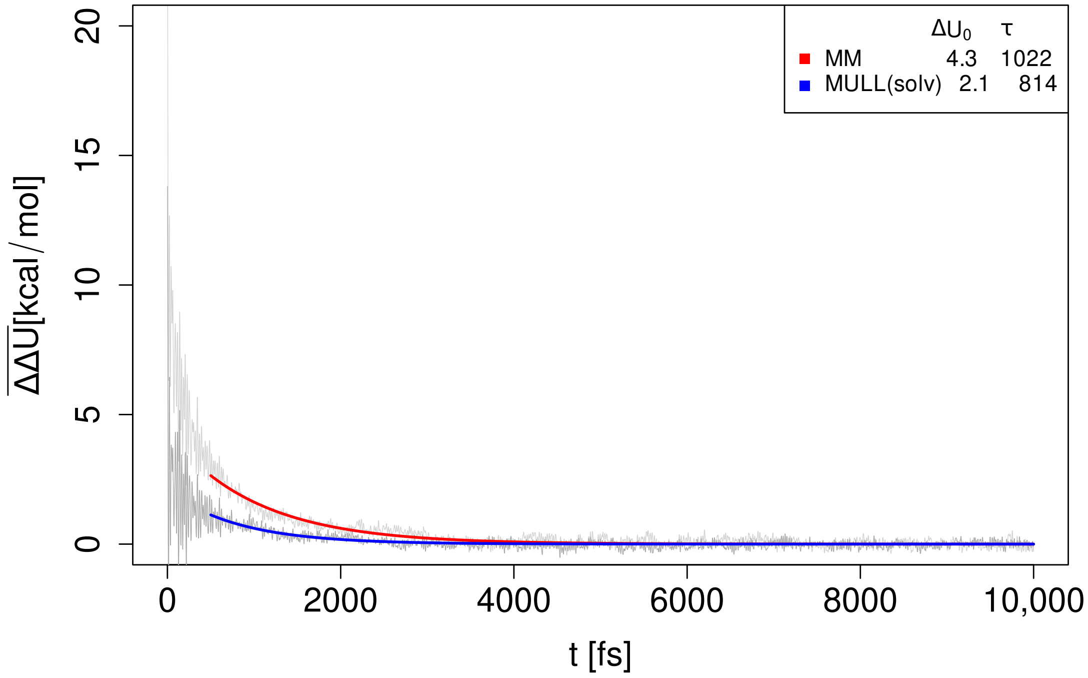

Figure S14. Relaxation of TIP3P Water for **8-t2** when switching from MM and MULL(solv) to SQM.

Table S2. Comparison of direct switches of 2 and 5 ps length, 2 and 5 ps CRO results, and the three approaches with an intermediate MULL state including standard error. These are the raw data for Figs. 4 and 5 of the main manuscript.

Compound	$\delta\Delta A_{solv}^{MM\rightarrow SQM}$	$\delta\Delta A_{solv}^{MM\leftrightarrow SQM}$	$\delta\Delta A_{solv}^{MM\rightarrow SQM}$	$\delta\Delta A_{solv}^{MM\leftrightarrow MULL(gas)\rightarrow SQM}$	$\delta\Delta A_{solv}^{MM\leftrightarrow MULL(solv)\rightarrow SQM}$	$\delta\Delta A_{solv}^{MM\leftrightarrow MULL(solv*)\rightarrow SQM}$
	JAR(fw)-CRO$_{Ref}$	JAR(fw)-CRO$_{Ref}$	CRO-CRO$_{Ref}$	BAR+JAR(fw)-CRO$_{Ref}$	BAR+JAR(fw)-CRO$_{Ref}$	BAR+JAR(fw)-CRO$_{Ref}$
N_{switch}	2000 fs	5000 fs	2000 fs	2000 fs	2000 fs	2000 fs
$N_{replicate}$	200	200	200	200	200	200
**1-t1**	0.35 ± 0.12	0.10 ± 0.08	-0.04 ± 0.05	0.21 ± 0.21	-0.13 ± 0.25	-0.06 ± 0.04
**1-t2**	0.01 ± 0.04	0.01 ± 0.04	0.02 ± 0.03	-0.01 ± 0.11	0.01 ± 0.07	0.01 ± 0.04
**2-t1**	-0.13 ± 0.13	-0.15 ± 0.12	0.14 ± 0.03	0.12 ± 0.08	0.01 ± 0.09	0.04 ± 0.04
**2-t2**	-0.10 ± 0.14	-0.05 ± 0.10	0.01 ± 0.05	-0.18 ± 0.35	0.03 ± 0.13	-0.07 ± 0.05
**3-t1**	0.22 ± 0.04	-0.11 ± 0.15	0.16 ± 0.04	0.01 ± 0.08	-0.02 ± 0.07	0.01 ± 0.04
**3-t2**	0.14 ± 0.18	0.06 ± 0.13	0.05 ± 0.07	0.09 ± 0.20	0.12 ± 0.15	-0.10 ± 0.07
**4-t1**	0.02 ± 0.07	-0.13 ± 0.11	0.02 ± 0.03	-0.09 ± 0.07	-0.07 ± 0.10	-0.20 ± 0.04
**4-t2**	0.08 ± 0.18	0.01 ± 0.10	-0.13 ± 0.06	0.05 ± 0.29	0.06 ± 0.12	-0.04 ± 0.06
**5-t1**	0.10 ± 0.05	-0.02 ± 0.03	0.03 ± 0.03	-0.02 ± 0.07	0.05 ± 0.07	0.01 ± 0.04
**5-t2**	1.16 ± 0.15	-0.02 ± 0.14	0.23 ± 0.07	0.32 ± 0.17	0.17 ± 0.17	0.02 ± 0.08
**6-t1**	0.04 ± 0.08	0.06 ± 0.06	-0.02 ± 0.04	-0.09 ± 0.09	-0.09 ± 0.06	-0.15 ± 0.04
**6-t2**	1.80 ± 0.42	0.76 ± 0.12	0.04 ± 0.16	0.20 ± 0.20	0.08 ± 0.19	-0.12 ± 0.11
**7-t1**	-0.09 ± 0.14	0.03 ± 0.06	0.01 ± 0.04	-0.08 ± 0.07	-0.04 ± 0.08	0.02 ± 0.05
**7-t2**	-0.22 ± 0.23	-0.22 ± 0.17	-0.22 ± 0.06	0.01 ± 0.37	-0.26 ± 0.29	-0.09 ± 0.06
**8-t1**	0.12 ± 0.05	0.04 ± 0.05	0.11 ± 0.04	-0.07 ± 0.11	-0.06 ± 0.08	-0.06 ± 0.04
**8-t2**	-0.33 ± 0.12	0.18 ± 0.05	-0.11 ± 0.05	0.08 ± 0.16	-0.09 ± 0.12	-0.11 ± 0.05
**Ala**	-0.14 ± 0.33	-0.04 ± 0.12	0.02 ± 0.06	-0.49 ± 0.37	-0.03 ± 0.26	-0.10 ± 0.12
**Ser**	0.13 ± 0.15	0.12 ± 0.12	-0.13 ± 0.07	0.03 ± 0.40	-0.26 ± 0.25	-0.01 ± 0.08
**MAD**	0.29 ± 0.15	0.12 ± 0.10	0.08 ± 0.05	0.12 ± 0.19	0.09 ± 0.14	0.07 ± 0.06

Table S3. $\Delta A_{gas}^{MM\rightarrow SQM}$ calculated with JAR (fw), JAR (bw), and CRO, as well as relevant convergence metrics, i.e., standard deviation $\sigma\Delta Ai$ for the 8 blocks, the average work $\overline{W}$, the standard deviation of W (σW), the one-sided Π_{CRO} and Π_{JAR} values, as well as percentage overlap for the distribution of work values between the JAR(fw) and JAR(bw) calculations.

	JAR(fw)							JAR(bw)							CRO		Overlap (%)	JAR(fw)-CRO
Compound	ΔA	Hyst	σ ΔAi	$\overline{W}$	σW	Π_{CRO}	Π_{JAR}	ΔA	Hyst	σ ΔAi	$\overline{W}$	σW	Π_{CRO}	Π_{JAR}	ΔA	$\sigma\,\Delta A$	W	$\delta\Delta A$
**1-t1**	-356.30	0.01	0.05	-356.19	0.31	0.13	2.46	356.19	0.01	0.07	358.92	2.99	4.11	0.28	-356.64	0.04	26.95	0.35
**1-t2**	-348.16	0.01	0.03	-348.10	0.25	2.41	2.61	348.14	0.01	0.02	348.22	0.33	2.84	2.54	-348.16	0.03	75.87	-0.01
**2-t1**	-873.45	0.01	0.03	-873.42	0.19	2.93	2.74	873.41	0.01	0.02	873.44	0.19	2.79	2.73	-873.43	0.03	90.10	-0.02
**2-t2**	-886.41	0.01	0.02	-886.39	0.15	3.40	2.79	886.37	0.01	0.02	886.39	0.15	2.67	2.80	-886.39	0.03	94.57	-0.02
**3-t1**	-870.41	0.01	0.02	-870.38	0.19	0.47	2.74	870.47	0.50	1.62	871.31	2.41	7.27	1.36	-870.48	0.03	82.21	0.07
**3-t2**	-867.93	0.01	0.02	-867.91	0.16	2.88	2.78	867.90	0.01	0.01	867.92	0.16	2.87	2.79	-867.92	0.03	87.11	-0.01
**4-t1**	-92.83	0.01	0.02	-92.81	0.18	2.77	2.32	92.79	0.01	0.29	92.82	0.19	2.19	2.29	-92.81	0.06	86.93	-0.02
**4-t2**	-105.54	0.01	0.03	-105.52	0.13	2.61	2.40	105.45	0.01	0.01	105.47	1.13	2.61	2.39	-105.50	0.06	61.56	-0.04
**5-t1**	-884.59	0.01	0.03	-884.57	0.18	2.42	2.32	884.57	0.01	0.03	884.60	0.17	2.38	2.33	-884.58	0.06	83.05	-0.01
**5-t2**	-872.67	0.01	0.05	-872.65	0.16	2.42	2.35	872.65	0.01	0.02	872.67	0.16	2.45	2.35	-872.66	0.06	84.63	-0.01
**6-t1**	-348.91	0.01	0.03	-348.87	0.24	2.73	2.67	348.92	0.01	0.02	348.96	0.21	2.58	2.68	-348.92	0.03	73.11	0.01
**6-t2**	-330.90	0.01	0.02	-330.88	0.13	2.87	2.83	330.89	0.01	0.01	330.90	0.13	2.91	2.84	-330.89	0.03	87.54	-0.01
**7-t1**	-737.05	0.01	0.04	-736.97	0.30	2.49	2.54	737.04	0.01	0.04	737.12	0.32	2.59	2.52	-737.04	0.03	64.73	-0.01
**7-t2**	-684.36	0.01	0.11	-683.67	1.10	2.21	1.53	684.41	0.01	0.06	684.79	0.60	1.27	1.91	-684.34	0.04	36.21	-0.02
**8-t1**	-203.44	0.01	0.06	-203.41	0.19	2.42	2.30	203.42	0.01	0.04	203.46	0.20	2.25	2.28	-203.43	0.06	80.87	-0.01
**8-t2**	-224.69	0.01	0.05	-224.67	0.17	2.32	2.33	224.67	0.01	0.03	224.69	0.17	2.48	2.33	-224.68	0.06	85.77	-0.01

Table S4. Detailed results for N_{switch}=2000 fs $N_{replicate}$=200 $\Delta A_{solv}^{MM\rightarrow SQM}$ in solution

	JAR(fw)							JAR(bw)							CRO		Overlap (%)	JAR(fw)-CRO
Compound	ΔA	Hyst	σ ΔAi	$\overline{W}$	σW	Π_{CRO}	Π_{JAR}	ΔA	Hyst	σ ΔAi	$\overline{W}$	σW	Π_{CRO}	Π_{JAR}	ΔA	σ ΔA	W	$\delta\Delta A$
**1-t1**	-357.92	0.07	0.33	-356.85	1.15	0.17	0.72	358.22	0.44	0.62	360.07	1.53	0.44	0.12	-358.31	0.12	15.61	0.39
**1-t2**	-348.34	0.01	0.10	-348.14	0.46	1.67	1.79	348.29	0.01	0.08	348.51	0.55	1.99	1.75	-348.31	0.07	55.96	-0.03
**2-t1**	-872.77	0.11	0.36	-872.32	0.57	1.65	1.38	872.50	0.01	0.13	872.71	0.51	1.97	1.77	-872.50	0.07	50.12	-0.27
**2-t2**	-891.36	0.12	0.39	-889.99	1.26	0.56	0.46	891.44	0.10	0.34	892.52	1.21	0.54	0.71	-891.27	0.12	20.81	-0.10
**3-t1**	-868.61	0.01	0.09	-868.37	0.53	1.07	1.72	868.59	0.10	0.43	869.15	1.03	2.02	1.24	-868.67	0.07	52.09	0.06
**3-t2**	-877.20	0.20	0.51	-875.19	1.64	-0.05	0.02	877.43	0.29	0.59	879.40	1.56	-0.04	0.04	-877.28	0.15	13.68	0.09
**4-t1**	-91.15	0.03	0.18	-90.90	0.50	1.14	1.69	91.08	0.09	0.39	91.57	0.96	2.11	1.33	-91.16	0.07	50.99	0.01
**4-t2**	-111.14	0.21	0.51	-109.69	1.42	0.34	0.41	111.22	0.37	0.59	112.91	1.34	0.25	0.24	-111.35	0.13	15.67	0.21
**5-t1**	-883.22	0.01	0.12	-883.03	0.48	1.48	1.82	883.33	0.01	0.09	883.60	0.66	1.80	1.66	-883.29	0.07	43.44	0.08
**5-t2**	-863.34	0.11	0.40	-861.67	1.73	-0.52	0.25	864.59	0.22	0.54	867.27	1.97	-0.37	-0.38	-864.27	0.17	10.95	0.93
**6-t1**	-345.60	0.03	0.21	-345.21	0.67	1.17	1.48	345.69	0.03	0.22	346.18	0.90	1.53	1.34	-345.65	0.08	39.68	0.05
**6-t2**	-313.78	0.74	1.16	-310.33	2.22	-1.69	-0.79	315.84	2.20	1.60	321.28	2.63	-1.65	-1.66	-315.55	0.43	2.00	1.76
**7-t1**	-727.52	0.13	0.39	-726.65	0.96	0.93	0.91	727.42	0.02	0.15	728.24	1.03	1.04	0.96	-727.42	0.09	27.88	-0.10
**7-t2**	-689.96	0.39	0.63	-687.80	1.94	0.31	-0.08	690.51	0.18	0.52	691.60	1.28	-0.07	0.70	-689.96	0.13	16.21	-0.01
**8-t1**	-200.32	0.01	0.13	-199.98	0.62	1.45	1.55	200.26	0.06	0.26	200.70	0.71	1.59	1.40	-200.33	0.08	44.61	0.01
**8-t2**	-226.31	0.08	0.33	-225.20	1.14	0.76	0.69	225.98	0.06	0.31	227.08	1.24	0.93	0.69	-226.10	0.10	29.24	-0.21
**Ala**	-456.14	0.45	0.92	-454.16	1.34	0.06	0.03	456.57	0.12	0.42	457.91	1.41	0.15	0.49	-455.98	0.15	10.98	-0.16
**Ser**	-534.85	0.13	0.41	-533.06	1.55	-0.17	0.16	535.46	0.14	0.47	537.44	1.89	-0.01	0.04	-535.10	0.16	15.06	0.26

Table S5. Detailed results for N_{switch}=5000 fs $N_{replicate}$=200 $\Delta A_{solv}^{MM\rightarrow SQM}$ in solution

	JAR(fw)							JAR(bw)							CRO_{Ref}		Overlap (%)	JAR(fw)-CRO
Compound	ΔA	Hyst	σ ΔAi	$\overline{W}$	σW	Π_{CRO}	Π_{JAR}	ΔA	Hyst	σ ΔAi	$\overline{W}$	σW	Π_{CRO}	Π_{JAR}	ΔA	σ ΔA	W	$\delta\Delta A$
**1-t1**	-358.18	0.03	0.22	-357.54	0.91	0.91	1.15	358.18	0.03	0.22	359.10	1.12	1.10	0.86	-358.28	0.09	31.51	0.10
**1-t2**	-348.34	0.01	0.08	-348.24	0.35	1.90	2.02	348.32	0.01	0.08	348.45	0.41	2.13	1.96	-348.34	0.07	68.09	-0.01
**2-t1**	-872.79	0.10	0.33	-872.46	0.45	1.15	1.56	872.61	0.09	0.44	872.96	0.83	2.51	1.53	-872.64	0.07	55.19	-0.15
**2-t2**	-891.31	0.05	0.26	-890.67	0.88	1.14	1.14	891.04	0.10	0.35	891.89	0.95	1.22	0.92	-891.27	0.09	36.60	-0.05
**3-t1**	-868.94	0.14	0.41	-868.51	0.49	0.86	1.41	868.88	0.25	0.79	869.44	1.09	2.17	1.24	-868.83	0.07	40.55	-0.11
**3-t2**	-877.28	0.09	0.34	-876.02	1.24	0.53	0.56	877.62	0.14	0.39	878.63	1.17	0.51	0.77	-877.33	0.12	18.59	0.06
**4-t1**	-91.30	0.07	0.30	-91.02	0.45	1.22	1.64	91.17	0.13	0.57	91.46	0.78	2.53	1.64	-91.18	0.07	55.76	-0.13
**4-t2**	-111.22	0.05	0.25	-110.34	1.08	0.91	0.89	111.28	0.05	0.27	112.09	1.03	0.88	0.97	-111.22	0.10	28.54	-0.01
**5-t1**	-883.34	0.01	0.07	-883.24	0.35	2.16	2.04	883.28	0.01	0.08	883.40	0.36	2.06	2.00	-883.32	0.06	68.08	-0.02
**5-t2**	-864.52	0.10	0.38	-863.11	1.34	0.36	0.44	864.67	0.13	0.47	865.99	1.41	0.47	0.51	-864.50	0.12	20.22	-0.02
**6-t1**	-345.58	0.02	0.14	-345.38	0.44	1.32	1.79	345.67	0.05	0.29	345.98	0.75	1.96	1.58	-345.63	0.07	42.08	0.06
**6-t2**	-314.82	0.31	0.61	-312.70	1.75	-0.58	-0.05	316.15	0.35	0.66	318.66	1.91	-0.51	-0.29	-315.59	0.18	8.47	0.76
**7-t1**	-727.40	0.02	0.16	-727.02	0.68	1.36	1.49	727.43	0.02	0.15	727.87	0.76	1.48	1.40	-727.43	0.08	41.75	0.03
**7-t2**	-689.96	0.18	0.48	-688.79	1.27	0.97	0.64	689.70	0.06	0.28	690.60	1.05	0.79	0.87	-689.74	0.10	29.56	-0.22
**8-t1**	-200.39	0.01	0.12	-200.21	0.45	1.21	1.83	200.47	0.03	0.21	200.79	0.90	2.19	1.58	-200.43	0.07	51.75	0.04
**8-t2**	-225.80	0.01	0.13	-225.46	0.66	1.14	1.55	226.08	0.02	0.18	226.57	0.83	1.37	1.33	-225.98	0.08	34.11	0.18
**Ala**	-456.03	0.09	0.33	-455.03	1.11	0.77	0.78	456.03	0.07	0.31	457.00	1.12	0.83	0.81	-456.00	0.10	24.98	-0.04
**Ser**	-534.85	0.06	0.31	-533.83	1.13	0.42	0.76	535.11	0.09	0.35	536.35	1.45	0.72	0.57	-534.97	0.11	22.77	0.12

Table S6. Detailed results for N_{switch}=2000 fs $N_{replicate}$=200 $\Delta A_{solv}^{MULL(gas)\rightarrow SQM}$ in solution

	JAR(fw)							JAR(bw)							CRO		Overlap (%)	JAR(fw)-CRO
Compound	ΔA	Hyst	$\sigma\ \Delta A$i	$\overline{W}$	σW	Π_{CRO}	Π_{JAR}	ΔA	Hyst	$\sigma\ \Delta A$i	$\overline{W}$	σW	Π_{CRO}	Π_{JAR}	ΔA	$\sigma\ \Delta A$	W	$\delta\Delta A$
**1-t1**	-300.97	0.09	0.34	-300.10	0.98	0.56	0.90	301.11	0.07	0.30	302.21	1.31	0.94	0.70	-301.03	0.10	26.00	0.07
**1-t2**	-325.98	0.02	0.18	-325.59	0.67	1.31	1.46	325.95	0.02	0.15	326.42	0.80	1.57	1.35	-325.98	0.08	43.10	-0.01
**2-t1**	-881.09	0.01	0.11	-880.96	0.39	1.60	1.93	881.24	0.05	0.28	881.45	0.52	1.82	1.77	-881.19	0.07	45.15	0.10
**2-t2**	-890.10	0.33	0.58	-888.55	1.25	0.43	0.34	889.83	0.11	0.40	891.33	1.36	0.48	0.37	-889.92	0.12	18.51	-0.18
**3-t1**	-874.39	0.01	0.12	-874.26	0.37	1.89	1.96	874.35	0.06	0.29	874.50	0.42	2.06	1.91	-874.38	0.07	55.87	-0.01
**3-t2**	-876.09	0.09	0.32	-874.77	1.27	0.37	0.51	875.97	0.14	0.47	877.63	1.43	0.48	0.25	-876.14	0.12	20.59	0.06
**4-t1**	-96.40	0.06	0.09	-96.28	0.36	1.88	1.96	96.35	0.05	0.30	96.48	0.41	2.19	1.96	-96.37	0.07	62.03	-0.03
**4-t2**	-109.36	0.16	0.47	-107.99	1.20	0.39	0.47	109.61	0.11	0.37	110.81	1.24	0.49	0.61	-109.36	0.12	16.88	0.01
**5-t1**	-886.239	0.01	0.10	-886.09	0.42	1.94	1.89	886.20	0.02	0.15	886.36	0.42	1.94	1.90	-886.22	0.07	58.46	-0.02
**5-t2**	-900.09	0.06	0.26	-899.10	1.05	0.40	0.79	900.45	0.10	0.37	901.73	1.33	0.64	0.54	-900.31	0.11	18.13	0.22
**6-t1**	-317.77	0.01	0.13	-317.61	0.40	1.83	1.89	317.65	0.04	0.24	317.81	0.47	2.29	1.88	-317.70	0.07	69.77	-0.07
**6-t2**	-317.66	0.07	0.27	-316.66	1.17	0.44	0.78	317.48	0.43	0.69	319.21	1.44	0.66	0.20	-317.85	0.11	22.24	0.18
**7-t1**	-753.88	0.01	0.09	-753.74	0.41	1.96	1.94	753.80	0.01	0.06	753.96	0.44	2.06	1.89	-753.85	0.07	69.80	-0.03
**7-t2**	-781.07	0.31	0.62	-778.59	2.16	0.04	-0.27	781.88	0.14	0.41	783.21	1.46	-0.31	0.50	-781.23	0.15	13.16	0.16
**8-t1**	-259.93	0.02	0.17	-259.78	0.38	2.01	1.90	259.82	0.01	0.06	259.91	0.34	2.29	2.06	-259.84	0.07	66.17	-0.09
**8-t2**	-268.62	0.04	0.25	-267.77	1.04	0.82	0.93	268.56	0.17	0.45	269.68	1.13	0.83	0.68	-268.72	0.10	23.42	0.10
**Ala**	-443.24	0.37	062	-441.70	1.20	0.60	0.34	443.12	0.11	0.39	444.09	1.18	0.62	0.81	-442.89	0.11	21.08	-0.35
**Ser**	-502.65	0.21	0.66	-501.29	1.28	0.30	0.47	502.89	0.10	0.37	504.23	1.50	0.49	0.50	-502.66	0.12	20.05	0.01

Table S7. Detailed results for energy correction term via FEP $\Delta A_{solv}^{MM\leftrightarrow MULL(gas)}$ in solution

	BAR		Cycle closure
Compound	ΔA	$\sigma\ \Delta A$	BAR+JAR(fw)-CRO_{Ref}
**1-t1**	-57.25	0.02	0.06
**1-t2**	-22.37	0.02	-0.01
**2-t1**	8.58	0.02	0.12
**2-t2**	-1.35	0.02	-0.18
**3-t1**	5.57	0.02	0.01
**3-t2**	-1.16	0.01	0.09
**4-t1**	5.14	0.02	-0.09
**4-t2**	-1.81	0.01	0.06
**5-t1**	2.90	0.01	-0.02
**5-t2**	35.92	0.03	0.32
**6-t1**	-27.95	0.02	-0.09
**6-t2**	2.27	0.04	0.20
**7-t1**	26.37	0.03	-0.08
**7-t2**	91.33	0.02	0.01
**8-t1**	59.42	0.02	-0.07
**8-t2**	42.72	0.02	0.08
**Ala**	-13.24	0.02	-0.49
**Ser**	-32.29	0.02	0.03

Table S8. Detailed results for N_{switch}=2000 fs $N_{replicate}$=200 $\Delta A_{solv}^{MULL(solv)\rightarrow SQM}$ in solution

	JAR(fw)							JAR(bw)							CRO		Overlap (%)	JAR(fw)-CRO
Compound	ΔA	Hyst	$\sigma\ \Delta A$i	$\overline{W}$	σW	Π_{CRO}	Π_{JAR}	ΔA	Hyst	$\sigma\ \Delta A$i	$\overline{W}$	σW	Π_{CRO}	Π_{JAR}	ΔA	$\sigma\ \Delta A$	W	$\delta\Delta A$
**1-t1**	-296.98	0.19	0.42	-296.29	0.80	0.89	1.10	296.95	0.05	0.29	297.72	1.08	1.27	1.00	-296.93	0.09	32.64	-0.04
**1-t2**	-290.29	0.01	0.08	-290.16	0.38	1.82	1.95	290.28	0.01	0.11	290.44	0.45	2.03	1.89	-290.29	0.07	58.94	0.01
**2-t1**	-872.19	0.01	0.13	-871.99	0.43	1.56	1.79	872.16	0.03	0.23	872.41	0.58	2.02	1.71	-872.18	0.07	52.42	-0.02
**2-t2**	-866.81	0.03	0.19	-866.42	0.67	1.30	1.47	866.77	0.02	0.15	867.31	0.83	1.50	1.28	-866.84	0.08	41.08	0.03
**3-t1**	-866.92	0.01	0.09	-866.76	0.39	1.58	1.88	866.92	0.10	0.43	867.13	0.56	2.10	1.77	-866.92	0.07	54.39	0.01
**3-t2**	-852.65	0.03	0.21	-852.07	0.83	0.99	1.22	852.76	0.07	0.28	853.53	0.95	1.11	1.02	-852.77	0.09	29.55	0.13
**4-t1**	-90.58	0.02	0.16	-90.44	0.35	1.66	1.92	90.52	0.08	0.41	90.66	0.46	2.43	1.95	-90.53	0.06	62.06	-0.05
**4-t2**	-87.81	0.02	0.17	-87.39	0.68	1.20	1.43	87.86	0.01	0.13	88.40	0.84	1.43	1.26	-87.86	0.08	34.61	0.06
**5-t1**	-882.67	0.01	0.10	-882.53	0.39	1.83	1.92	882.73	0.01	0.11	882.85	0.38	1.93	2.00	-882.68	0.07	52.09	0.02
**5-t2**	-877.79	0.05	0.26	-877.25	0.80	0.99	1.26	878.04	0.02	0.17	878.66	0.93	1.17	1.17	-877.92	0.09	30.33	0.13
**6-t1**	-319.78	0.01	0.07	-319.64	0.36	1.63	1.93	319.71	0.06	0.35	319.86	0.50	2.48	1.89	-319.73	0.06	66.43	-0.05
**6-t2**	-306.52	0.05	0.25	-305.44	1.23	0.52	0.71	306.26	0.48	0.67	308.01	1.30	0.55	0.19	-306.72	0.11	21.47	0.20
**7-t1**	-728.78	0.01	0.10	-728.66	0.37	1.95	1.97	728.77	0.01	0.09	728.90	0.40	2.01	1.95	-728.78	0.07	60.67	-0.01
**7-t2**	-744.16	0.13	0.48	-742.17	2.17	1.12	0.03	743.78	0.51	0.70	745.00	0.91	0.08	0.59	-744.03	0.13	21.91	-0.13
**8-t1**	-252.61	0.01	0.10	-252.52	0.31	2.05	2.05	252.49	0.01	0.06	252.56	0.29	2.69	2.13	-252.53	0.06	87.34	-0.08
**8-t2**	-244.81	0.02	0.18	-244.54	0.54	1.56	1.67	244.69	0.01	0.10	245.03	0.68	1.87	1.56	-244.77	0.07	52.40	-0.04
**Ala**	-422.18	0.13	0.42	-421.59	0.83	1.10	1.20	422.25	0.05	0.25	422.90	0.88	1.16	1.15	-422.23	0.09	29.88	0.05
**Ser**	-483.98	0.14	0.40	-483.15	0.97	0.77	0.94	483.85	0.04	0.22	484.78	1.26	1.19	0.85	-483.87	0.09	33.84	-0.11

Table S9. Detailed results for energy correction term via FEP $\Delta A_{solv}^{MM\leftrightarrow MULL(solv)}$ in solution

	BAR		Cycle closure
Compound	ΔA	$\sigma\ \Delta A$	BAR+JAR(fw)-CRO_{Ref}
**1-t1**	-61.43	0.05	-0.13
**1-t2**	-58.04	0.04	0.02
**2-t1**	-0.46	0.01	-0.01
**2-t2**	-24.43	0.03	0.03
**3-t1**	-1.96	0.02	-0.04
**3-t2**	-24.52	0.03	0.17
**4-t1**	-0.68	0.01	-0.08
**4-t2**	-23.37	0.03	0.05
**5-t1**	-0.61	0.01	0.04
**5-t2**	13.41	0.07	0.11
**6-t1**	-25.96	0.02	-0.11
**6-t2**	-8.96	0.22	0.11
**7-t1**	1.30	0.02	-0.05
**7-t2**	54.15	0.03	-0.26
**8-t1**	52.12	0.03	-0.06
**8-t2**	18.71	0.03	-0.12
**Ala**	-33.84	0.04	-0.03
**Ser**	-51.25	0.04	-0.26

Table S10. Detailed results for N_{switch}=2000 fs $N_{replicate}$=200 $\Delta A_{solv}^{MULL(solv*)\rightarrow SQM}$ in solution

	JAR(fw)							JAR(bw)							CRO		Overlap (%)	JAR(fw)-CRO
Compound	ΔA	Hyst	$\sigma\ \Delta A$i	$\overline{W}$	σW	Π_{CRO}	Π_{JAR}	ΔA	Hyst	$\sigma\ \Delta A$i	$\overline{W}$	σW	Π_{CRO}	Π_{JAR}	ΔA	$\sigma\ \Delta A$	W	$\delta\Delta A$
**1-t1**	-255.56	0.01	0.05	-255.46	0.33	1.69	2.04	255.59	0.01	0.05	255.76	0.53	2.16	1.86	-255.59	0.06	57.14	0.04
**1-t2**	-280.90	0.01	0.11	-280.76	0.40	1.69	1.91	280.87	0.01	0.09	281.10	0.55	2.04	1.74	-280.91	0.07	55.25	0.01
**2-t1**	-871.50	0.01	0.12	-871.36	0.37	2.03	1.94	871.43	0.01	0.08	871.51	0.31	2.21	2.10	-871.43	0.06	63.71	-0.07
**2-t2**	-845.45	0.01	0.06	-845.40	0.24	2.33	2.22	845.40	0.01	0.04	845.44	0.23	2.35	2.23	-845.42	0.06	86.69	-0.02
**3-t1**	-867.80	0.01	0.10	-867.70	0.33	1.87	2.03	867.70	0.02	0.18	867.80	0.38	2.57	2.03	-867.74	0.06	81.16	-0.05
**3-t2**	-821.91	0.01	0.06	-821.85	0.27	2.25	2.16	821.82	0.01	0.05	821.89	0.29	2.46	2.12	-821.87	0.06	88.84	-0.04
**4-t1**	-90.98	0.02	0.17	-90.83	0.38	1.64	1.88	90.82	0.04	0.27	90.95	0.45	2.82	1.94	-90.87	0.07	74.48	-0.11
**4-t2**	-65.33	0.01	0.05	-65.29	0.21	2.24	2.25	65.30	0.01	0.04	65.35	0.23	2.40	2.24	-65.32	0.06	80.71	-0.02
**5-t1**	-879.94	0.01	0.10	-879.84	0.32	1.90	2.02	879.92	0.01	0.08	880.02	0.38	2.23	2.03	-879.92	0.06	63.06	-0.02
**5-t2**	-870.07	0.01	0.06	-870.01	0.25	2.20	2.18	870.02	0.01	0.04	870.08	0.27	2.34	2.17	-870.05	0.06	80.10	-0.02
**6-t1**	-315.73	0.01	0.06	-315.57	0.37	1.55	1.88	315.56	0.01	0.11	315.68	0.43	3.12	2.00	-315.61	0.06	77.97	-0.12
**6-t2**	-279.51	0.01	0.06	-279.48	0.20	2.24	2.27	279.50	0.01	0.04	279.54	0.21	2.36	2.28	-279.50	0.06	77.40	-0.01
**7-t1**	-739.64	0.01	0.06	-739.57	0.28	2.04	2.14	739.67	0.01	0.07	739.75	0.32	2.08	2.09	-739.66	0.06	61.21	0.02
**7-t2**	-706.62	0.03	0.22	-704.88	2.05	1.44	0.20	706.81	0.05	0.27	707.35	0.65	0.16	1.27	-706.56	0.12	24.94	-0.06
**8-t1**	-253.13	0.01	0.05	-253.03	0.31	2.07	2.03	253.03	0.01	0.04	253.08	0.23	2.63	2.22	-253.05	0.06	71.13	-0.08
**8-t2**	-226.64	0.01	0.05	-226.60	0.22	2.27	2.24	226.61	0.01	0.04	226.65	0.23	2.37	2.24	-226.62	0.06	86.52	-0.02
**Ala**	-393.52	0.16	0.39	-393.08	0.68	1.25	1.40	393.59	0.02	0.16	394.02	0.78	1.48	1.41	-393.52	0.08	38.17	0.01
**Ser**	-449.92	0.06	0.29	-449.52	0.70	0.98	1.45	450.01	0.04	0.23	450.69	1.05	1.49	1.11	-450.02	0.08	37.71	0.10

Table S11. Detailed results for energy correction term via FEP $\Delta A_{solv}^{MM\leftrightarrow MULL(solv*)}$ in solution

	BAR		Cycle closure
Compound	ΔA	$\sigma\ \Delta A$	BAR+JAR(fw)-CRO$_{Ref}$
**1-t1**	-102.78	0.03	-0.06
**1-t2**	-67.42	0.02	0.01
**2-t1**	-1.10	0.01	0.04
**2-t2**	-45.89	0.03	-0.07
**3-t1**	-1.02	0.03	0.01
**3-t2**	-55.53	0.03	-0.11
**4-t1**	-0.39	0.01	-0.20
**4-t2**	-45.93	0.03	-0.04
**5-t1**	-3.38	0.02	0.01
**5-t2**	5.59	0.03	0.02
**6-t1**	-30.05	0.02	-0.15
**6-t2**	-36.20	0.05	-0.12
**7-t1**	12.22	0.02	0.02
**7-t2**	16.80	0.02	-0.09
**8-t1**	52.64	0.02	-0.06
**8-t2**	0.55	0.02	-0.11
**Ala**	-62.58	0.03	-0.10
**Ser**	-85.06	0.03	-0.01

Table S12. Compound, Root Mean Square Deviation of atomic charges relative to SQM for MM-SQM($RMSD_q[C]_{MM}$), MULL(gas)-SQM($RMSD_q[C]_{Mull(gas)}$) and MULL(solv)-SQM($RMSD_q[C]_{Mull(solv)}$). Differential dipole moment MM-SQM($\Delta\mu_{MM}$), differential dipole moment MULL(gas)-SQM($\Delta\mu_{MULL(gas)}$), differential dipole moment MULL(solv)-SQM($\Delta\mu_{MULL(solv)}$), angle between dipole moments relative to SQM for MM, MULL(gas) and MULL(solv))

Compound	$RMSD_q$[e]			$\Delta\mu$[D]			θ_{SQM}[°]		
	MM	$MULL(gas)$	$MULL(solv)$	MM	$MULL(gas)$	$MULL(solv)$	MM	$MULL(gas)$	$MULL(solv)$
**1-t1**	0.16	0.09	0.06	4.65	2.87	2.61	52.4	17.3	25.7
**1-t2**	0.08	0.06	0.01	0.15	0.56	0.09	2.2	2.0	1.3
**2-t1**	0.06	0.02	0.01	1.67	0.57	0.91	20.7	13.8	15.9
**2-t2**	0.16	0.09	0.05	3.92	5.31	2.72	1.0	0.3	1.7
**3-t1**	0.07	0.02	0.01	1.45	0.24	0.98	31.4	0.3	23.1
**3-t2**	0.16	0.09	0.06	5.91	5.63	3.78	18.3	9.4	10.0
**4-t1**	0.07	0.02	0.01	1.42	0.21	0.80	17.7	8.0	13.4
**4-t2**	0.20	0.11	0.07	3.72	4.49	2.52	12.5	6.4	7.8
**5-t1**	0.05	0.02	0.01	1.89	0.28	1.00	37.5	18.7	25.2
**5-t2**	0.18	0.08	0.06	6.74	4.14	3.21	49.3	9.1	24.3
**6-t1**	0.13	0.02	0.02	1.04	0.42	0.58	32.3	8.0	19.3
**6-t2**	0.29	0.11	0.10	8.10	4.17	4.22	58.3	10.6	31.0
**7-t1**	0.14	0.02	0.02	3.35	0.78	0.99	86.4	13.3	24.4
**7-t2**	0.17	0.11	0.05	6.96	6.54	3.45	8.1	1.5	4.7
**8-t1**	0.16	0.02	0.02	1.50	0.36	0.62	85.1	1.3	48.1
**8-t2**	0.24	0.10	0.05	2.94	3.61	2.00	2.2	12.3	6.5
**Ala**	0.12	0.07	0.04	2.82	2.11	1.16	5.0	0.4	0.7
**Ser**	0.13	0.07	0.04	2.61	1.67	1.01	16.7	3.5	4.2
**MAD**	0.14	0.06	0.04	3.38	2.44	1.81	29.8	7.6	16.0

Table S13. Resulting parameters from mono-exponential fitting of the relaxation process for a set of compounds; ΔU_0 represents the initial value, τ is the resulting time constant of the fit, ΔU_{2000} is the difference in energy to the baseline ($\approx$0 kcal/mol) after 2000 fs of relaxation time.

	MM			MULL(solv)		
Compound	ΔU_0 [kcal/mol]	τ [fs^{-1}]	ΔU_{2000} [kcal/mol]	ΔU_0 [kcal/mol]	τ [fs^{-1}]	ΔU_{2000} [kcal/mol]
**4-t2**	8.2	996	1.1	3.5	1052	0.5
**5-t2**	10.6	1075	1.7	4.2	908	0.5
**6-t2**	19.6	744	1.3	6.3	933	0.7
**8-t2**	4.3	1022	0.6	2.1	814	0.2

1. Crooks, G.E. Path-ensemble averages in systems driven far from equilibrium. *Phys. Rev. E* **2000**, *61*, 2361. https://doi.org/10.1103/PhysRevE.61.2361.
2. Jarzynski, C. Nonequilibrium equality for free energy differences. *Phys. Rev. Lett.* **1997**, *78*, 2690. https://doi.org/10.1103/PhysRevLett.78.2690.
3. Kearns, F.L.; Warrensford, L.; Boresch, S.; Woodcock, H.L. The Good, the Bad, and the Ugly: "HiPen", a New Dataset for Validating (S)QM/MM Free Energy Simulations. *Molecules* **2019**, *24*. https://doi.org/10.3390/molecules24040681.
4. Wood, R.H.; Muhlbauer, W.C.F.; Thompson, P.T. Systematic errors in free energy perturbation calculations due to a finite sample of configuration space: sample-size hysteresis. *J. Phys. Chem.* **1991**, *95*, 6670–6675, [https://doi.org/10.1021/j100170a054]. https://doi.org/10.1021/j100170a054.
5. Wu, D.; Kofke, D.A. Model for small-sample bias of free-energy calculations applied to Gaussian-distributed nonequilibrium work measurements. *J. Chem. Phys.* **2004**, *121*, 8742–8747. https://doi.org/10.1063/1.1806413.
